# Supplementary material for: KNexPHENIX: A PHENIX-Based Workflow for Improving Cryo-EM and Crystallographic Structural Models
Source: J Chem Inf Model. 2026 Jun 9;66(12):6814–20. doi: 10.1021/acs.jcim.5c02404 (PMC13292208; doi:10.1021/acs.jcim.5c02404)
Supplement: Supplementary file 1 [file ci5c02404_si_001.pdf]

SUPPLEMENTARY INFORMATION

KNexPHENIX: A PHENIX-Based Workflow for Improving Cryo-EM and Crystallographic Structural Models

Suparno Nandi[a,*] and Graeme L. Conn[a,*]

[a]Department of Biochemistry, Emory University School of Medicine, Atlanta, GA 30322, USA

*Address correspondence to: nandisuparno@gmail.com or gconn@emory.edu

This file contains:

Supplementary Figures S1-S5

Supplementary Tables S1-S9

Supplementary Methods
- Default PHENIX refinement for cryo-EM and X-ray crystal structures
- Model refinement in REFMAC

Supplementary Results
- Motivation for stage and parameter selection in the KNexPHENIX refinement pipeline
- Typical duration and efficiency of KNexPHENIX refinement
- Analyses of the effect of variation of stages and parameters in KNexPHENIX

Supplementary References

Case Study 1: PI3Kalpha H1047R cryo-EM structure (PDB code 8GUB)

Case study 2: Monoubiquitinated PCNA X-ray crystal structure (PDB code 3L0W)

KNexPHEX “How-to” guide (**Workflows 1-4** with PHENIX screenshots)

Supplementary Figures

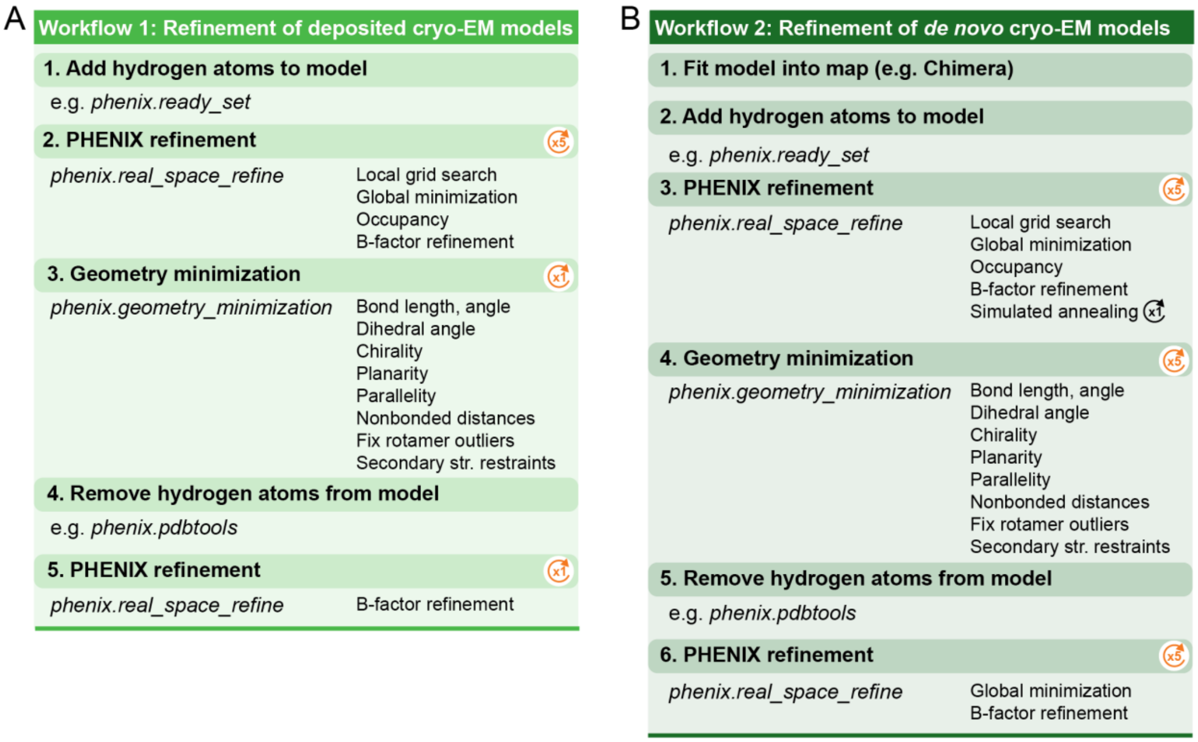

Fig. S1. KNexPHENIX workflows for refinement of structures determined by cryoEM. ***A***, Workflow 1 for refinement of existing cryo-EM models against their corresponding maps. ***B***, Workflow 2 for refinement of new cryo-EM models in the process of *de novo* structure determination.

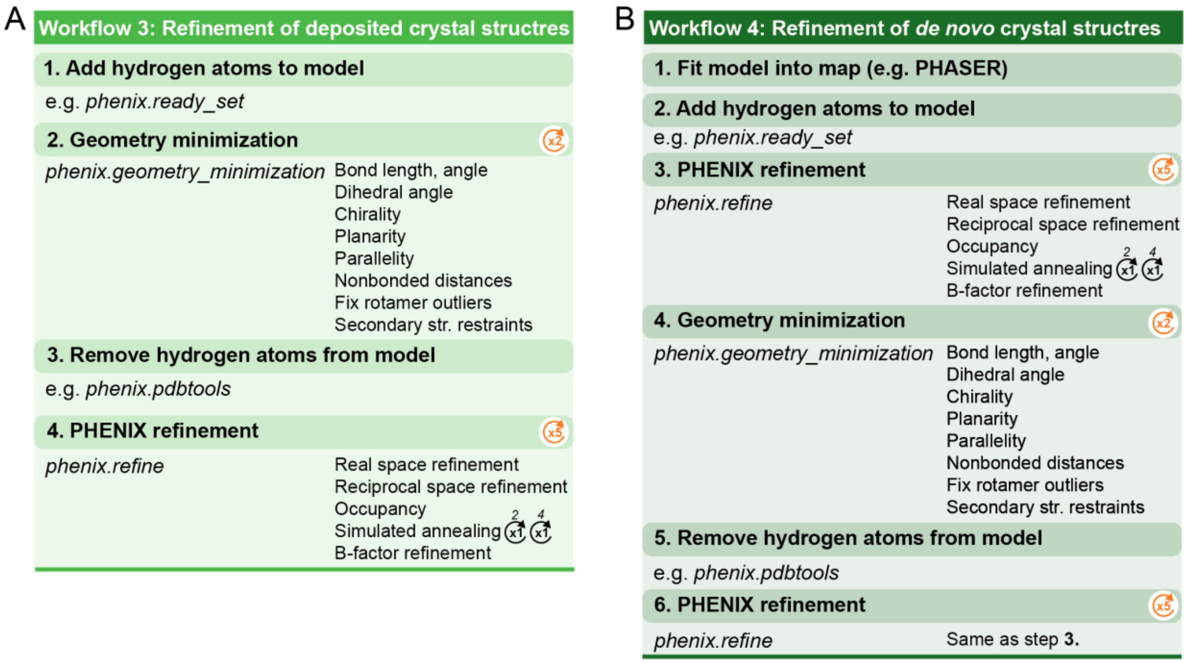

Fig. S2. KNexPHENIX workflows for refinement of structures determined by X-ray crystallography. ***A***, Workflow 3 for refinement of existing X-ray crystal structures against their corresponding maps. ***B***, Workflow 4 for refinement of new X-ray crystal structures in the process of structure determination by MR.

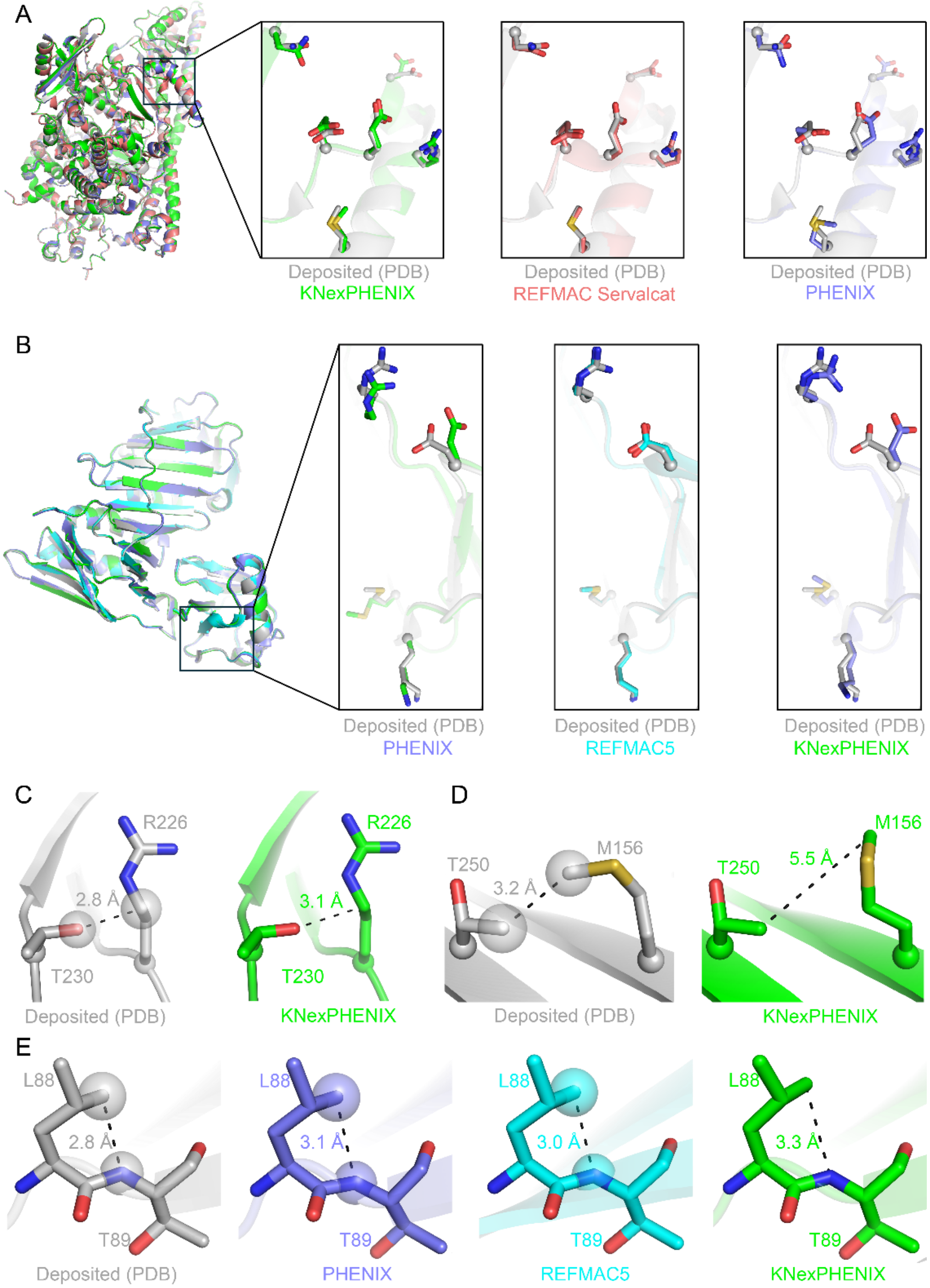

Fig. S3. KNexPHENIX refines local side chain geometry and resolves atomic clashes without compromising fit to the map. ***A,*** Protein backbone conformation is consistent across the structures refined using KNexPHENIX (green), REFMAC Servalcat (red), PHENIX (blue) for the PI3Kalpha H1047R variant determined by cryo-EM (PDB code 8GUB) and all align well with the original PDB model (gray). Sidechain position differences compared to the deposited structure are observed in the structures refined with KNexPHENIX and PHENIX, whereas REFMAC Servalcat leaves them essentially unchanged. ***B,*** The monoubiquitinated PCNA crystal structure (PDB code 3L0W) refined by KNexPHENIX (green), REFMAC5 (cyan), and PHENIX (blue) shows overall structural similarity with the PDB model (gray). REFMAC5 does not change the position of the side chains, while both PHENIX and KNexPHENIX produce more substantial movements. ***C,*** In the KNexPHENIX-refined R220A metBJFIXL HEME domain crystal structure (PDB code 1Y28), a shift of R226 resolves a steric clash with T230 present in the original PDB structure. ***D,*** Steric hindrance is also avoided by rotation of the M156 side chain from T250 by KNexPHENIX refinement. ***E,*** PHENIX and REFMAC5 do not relieve a steric clash between L88 and T89 observed in the monoubiquitinated PCNA crystal structure (PDB code 3L0W) which is successfully resolved by KNexPHENIX.

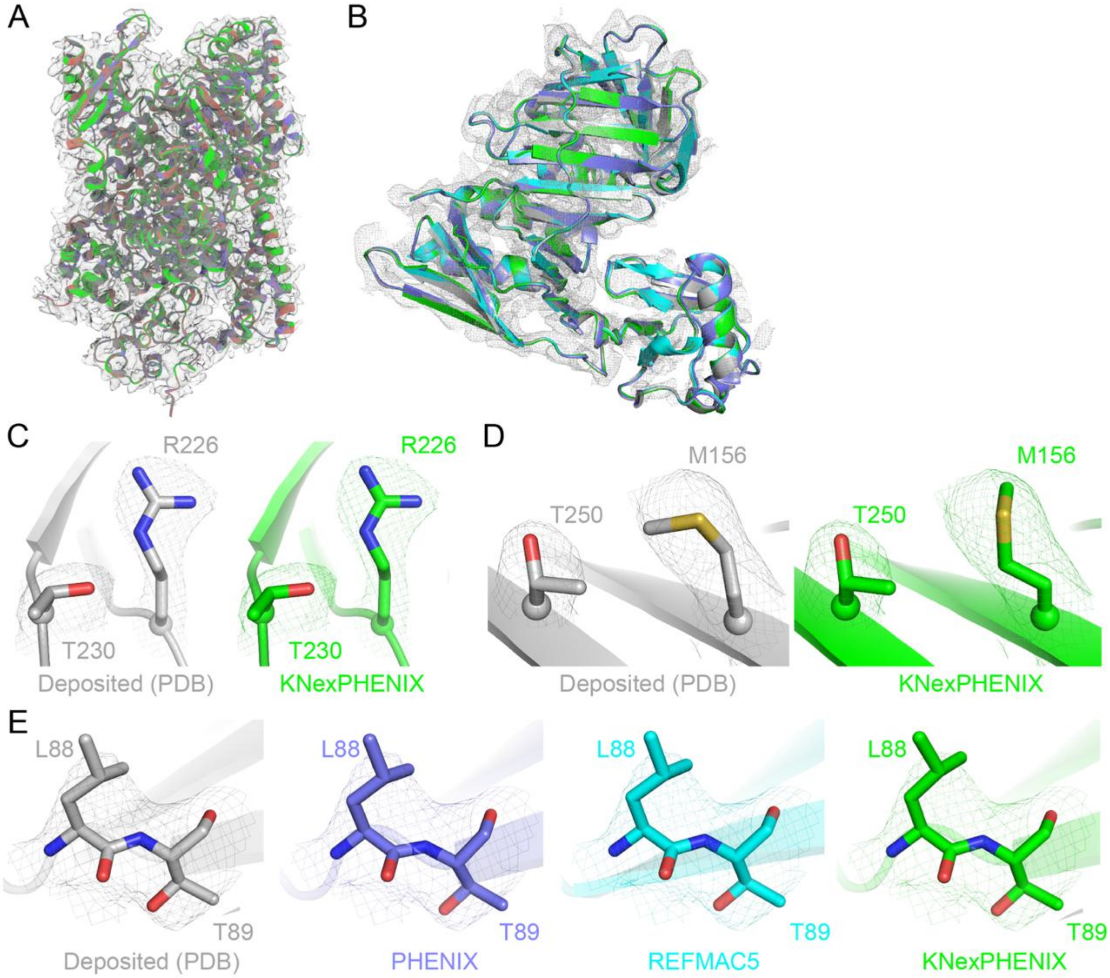

Fig. S4. KNexPHENIX maintains a good model fit to map while improving model quality. ***A,*** The PHENIX (blue), Servalcat (red), and KNexPHENIX (green) refined PI3Kalpha variant H1047R cryo-EM structure aligns well with the PDB model (gray) and fits well into the map. ***B,*** Similarly, monoubiquitinated PCNA refined by KNexPHENIX, PHENIX, REFMAC5 (cyan) also fits well into the map and is in overall good agreement with the deposited structure (PDB). KNexPHENIX refinement does not shift the residues significantly to affect map fit in the ***C-D,*** R220A metBJFIXL HEME domain crystal structure, or in ***E,*** the monoubiquitinated PCNA crystal structure. Related to **Fig. S3**.

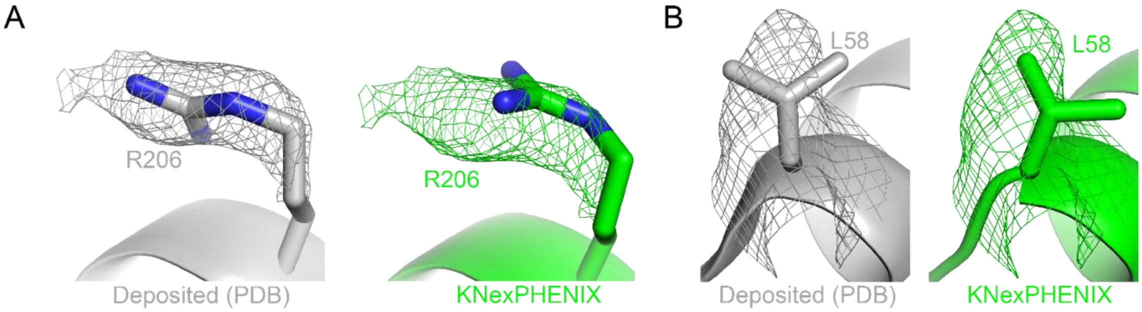

Fig. S5. Overcorrection of rotamer outliers by KNexPHENIX results in poor model-to-map-fit. ***A,*** KNexPHENIX (green) refinement of the deposited crystal structure (gray) of the metBJFIXL variant (PDB code 1Y28) results in correction of the rotamer outlier of R206 causing disagreement between model and map. ***B,*** Likewise, L58 in the deposited KDEL receptor variant structure (gray, PDB code 8APY) is well supported by the density, which is overcorrected by KNexPHENIX (green), shifting it outside the map.

Supplementary Tables

Table S1: Structures used for KNexPHENIX benchmarking against other refinement approaches.

	PDB code	Starting Model Use	Composition	Size (kDa)	Resolution (Å)
Cryo-EM	9E0N	*De novo* only	Nucleoprotein complex	2,280	3.24
	5AN9	Re-refine & *de novo*	Nucleoprotein complex	1,419	3.30
	8ETH	Re-refine & *de novo*	Nucleoprotein complex	2,428	3.80
	5H1S	Re-refine & *de novo*	Nucleoprotein complex	1,445	3.50
	6OF4	Re-refine & *de novo*	Protein	227	3.20
	5A1A	Re-refine & *de novo*	Protein	476	2.20
	6JO5	Re-refine & *de novo*	Protein	765	2.90
	8GUB	Re-refine & *de novo*	Protein	212	2.73
	8GUD	Re-refine & *de novo*	Protein	128	2.62
	6YEZ	Re-refine & *de novo*	Protein	573	2.70
	8ASW	Re-refine only	Nucleoprotein complex	483	3.96
	7UN3	Re-refine only	Protein	478	3.50
	7W0L	Re-refine only	Protein	223	3.57
	7W0P	Re-refine only	Protein	176	3.16
X-ray crystallography	1Y28	Re-refine & *de novo*	Protein	15	2.10
	3L0W	Re-refine & *de novo*	Protein	38	2.80
	6KBI	Re-refine & *de novo*	Protein	140	3.00
	8F0V	Re-refine & *de novo*	Protein	19	2.95
	8HUK	Re-refine & *de novo*	Protein	64	2.98
	1YNS	Re-refine & *de novo*	Protein	29	1.70
	1FT2	Re-refine & *de novo*	Protein	83	3.40
	1D8U	Re-refine & *de novo*	Protein	38	2.35
	6SZW	Re-refine & *de novo*	Protein-protein complex	80	3.14
	8APY	Re-refine & *de novo*	Protein-protein complex	38	2.34
	3UZ0	Re-refine only	Protein-protein complex	63	2.82
	3JWR	Re-refine only	Protein-protein complex	82	2.99
	1ZOY	Re-refine only	Protein-protein complex	127	2.40
	4YJ5	Re-refine only	Protein	229	2.41
	1R30	Re-refine only	Protein	85	3.40
	1M52	Re-refine only	Protein	69	2.60

Table S2: MolProbity score (MS) and CC_{mask} calculated from 13 cryo-EM structures deposited in the PDB and after their refinement using PHENIX, REFMAC Servalcat, and KNexPHENIX.

PDB code	PDB (deposited)[a]		PHENIX[a]		REFMAC Servalcat[a]		KNexPHENIX[a]	
	MS	CC_{mask}	MS	CC_{mask}	MS	CC_{mask}	MS	CC_{mask}
5AN9	2.02	0.81	2.38	0.80	3.17	0.84	1.79	0.78
8ETH	1.89	0.61	2.20	0.64	3.04	0.70	1.52	0.61
6OF4	2.65	0.82	2.16	0.81	3.51	0.90	1.52	0.78
7UN3	2.00	0.63	2.03	0.68	3.61	0.80	1.50	0.62
8ASW	2.35	0.78	2.77	0.78	3.81	0.88	1.73	0.74
7W0L	2.15	0.71	2.31	0.78	3.61	0.88	1.77	0.74
5A1A	1.97	0.76	2.28	0.79	1.82	0.80	1.32	0.76
8GUB	2.35	0.65	2.45	0.78	2.82	0.82	1.24	0.76
6JO5	1.87	0.84	2.17	0.89	2.65	0.90	1.63	0.81
6YEZ	2.35	0.87	2.17	0.91	2.72	0.93	1.93	0.89
5H1S	3.24	0.77	2.80	0.82	3.34	0.89	2.33	0.77
8GUD	2.20	0.56	2.14	0.54	3.72	0.64	1.50	0.53
7W0P	2.05	0.59	2.24	0.70	3.14	0.83	1.64	0.67
Mean	*2.24*	*0.72*	*2.32*	*0.76*	*3.15*	*0.83*	*1.65*	*0.73*

[a]Data used to generate plots shown in **Fig. 1A,B**.

Table S3: MolProbity score (MS) and CC_{mask} calculated from 10 cryo-EM structures deposited in the PDB and after their refinement using PHENIX, REFMAC Servalcat, CERES, and KNexPHENIX.

PDB code	PDB (deposited)[a]		PHENIX[a]		REFMAC Servalcat[a]		CERES[a]		KNexPHENIX[a]	
	MS	CC_{mask}	MS	CC_{mask}	MS	CC_{mask}	MS	CC_{mask}	MS	CC_{mask}
5AN9	2.02	0.81	2.38	0.80	3.17	0.84	2.25	0.81	1.79	0.78
8ETH	1.89	0.61	2.20	0.64	3.04	0.70	1.86	0.60	1.52	0.61
6OF4	2.65	0.82	2.16	0.81	3.51	0.90	1.93	0.81	1.52	0.78
7UN3	2.00	0.63	2.03	0.68	3.61	0.80	1.97	0.69	1.50	0.62
8ASW	2.35	0.78	2.77	0.78	3.81	0.88	2.21	0.78	1.73	0.74
7W0L	2.15	0.71	2.31	0.78	3.61	0.88	2.01	0.77	1.77	0.74
5A1A	1.97	0.76	2.28	0.79	1.82	0.80	1.95	0.79	1.32	0.76
8GUB	2.35	0.65	2.45	0.78	2.82	0.82	2.00	0.79	1.24	0.76
5H1S	3.24	0.77	2.80	0.82	3.72	0.64	2.41	0.83	2.33	0.77
8GUD	2.20	0.56	2.14	0.54	3.14	0.83	2.26	0.56	1.50	0.53
Mean	*2.28*	*0.71*	*2.35*	*0.74*	*3.23*	*0.81*	*2.09*	*0.74*	*1.62*	*0.71*

[a]Data used to generate plots shown in **Fig. 1C,D**.

Table S4: MolProbity score (MS) and CC_{mask} calculated from *de novo* refinement of 10 cryo-EM structures by PHENIX, REFMAC Servalcat, and KNexPHENIX using maps extracted from the PDB.

PDB code[a]	Starting Model	PHENIX[b]		REFMAC Servalcat[b]		KNexPHENIX[b]	
		MS	CC_{mask}	MS	CC_{mask}	MS	CC_{mask}
8GUD	8GUA	2.22	0.50	3.39	0.61	1.17	0.45
8GUB	8DD4	1.93	0.75	2.91	0.81	1.08	0.67
6OF4	6OF2	2.09	0.79	3.45	0.89	1.29	0.74
5A1A	6CVM	2.11	0.80	1.27	0.76	1.14	0.73
5H1S	5X8T	2.66	0.83	3.26	0.89	1.94	0.78
6JO5	7BGI	1.39	0.86	1.96	0.88	0.78	0.79
8ETH	8EV3	2.10	0.62	2.95	0.70	1.51	0.60
5AN9	6QKL	2.64	0.83	3.17	0.85	1.76	0.80
6YEZ	6ZOO	1.71	0.92	2.24	0.92	0.90	0.86
9E0N	5ZEB	2.53	0.86	3.43	0.89	1.85	0.83
	Mean	*2.14*	*0.78*	*2.80*	*0.82*	*1.34*	*0.73*

[a]Deposition corresponding to the map used for refinement.
[b]Data used to generate plots shown in **Fig. 2**.

Table S5: Comparison of MolProbity score (MS), R_{work}, R_{free}, ΔR (R_{free}-R_{work}), and/or CC_{mask} from *de novo* KNexPHENIX refinement of two cryo-EM and two crystal structures using a starting model from AlphaFold (KNexPHENIX-AF), Boltz2 (KNexPHENIX-Boltz2), RoseTTAFold3 (KNexPHENIX-RF3), and an initial model selected from the PDB.

Cryo-EM

PDB code[a]	KNexPHENIX[b]		KNexPHENIX-AF		KNexPHENIX-Boltz2		KNexPHENIX-RF3	
	MS	CC_{mask}	MS	CC_{mask}	MS	CC_{mask}	MS	CC_{mask}
8GUD	1.17	0.45	0.77	0.44	0.87	0.42	0.96	0.42
5A1A[c]	1.14	0.73	1.04	0.74	-	-	-	-

X-ray Crystallography

PDB code[a]	KNexPHENIX[b]				KNexPHENIX-AF				KNexPHENIX-Boltz2				KNexPHENIX-RF3			
	MS	R_{work}	R_{free}	ΔR	MS	R_{work}	R_{free}	ΔR	MS	R_{work}	R_{free}	ΔR	MS	R_{work}	R_{free}	ΔR
1Y28[d]	0.91	28.4	30.6	2.20	1.29	29.7	32.5	2.80	1.39	31.9	32.8	0.90	1.17	33.5	34.7	1.20
8F0V	1.64	34.4	39.0	4.60	2.15	29.8	40.4	10.6	2.01	28.6	36.9	8.30	1.58	37.5	41.9	4.40

[a]Deposition corresponding to the map used for refinement.
[b]Original model selected from the PDB (see **Tables S4** and **S7**).
[c]Boltz2 and RF3 could not predict a starting model for 5A1A as the total number of residues is >3,500.
[d]The AF model corresponding to 1Y28 was truncated to remove residues without corresponding density prior to refinement.

Table S6: MolProbity score (MS), R_{work}, R_{free}, and ΔR (R_{free}-R_{work}) calculated from 16 crystal structures deposited in the PDB and after their refinement using PHENIX, REFMAC5, and KNexPHENIX.

PDB code	PDB (deposited)[a]				PHENIX[a]				REFMAC5[a]				KNexPHENIX[a]			
	MS	R_{work}	R_{free}	ΔR	MS	R_{work}	R_{free}	ΔR	MS	R_{work}	R_{free}	ΔR	MS	R_{work}	R_{free}	ΔR
1Y28	2.75	23.4	26.9	3.5	1.70	20.8	24.3	3.5	2.45	20.2	23.4	3.2	1.45	21.3	23.8	2.5
3UZ0	3.29	20.9	26.8	5.9	2.78	21.1	27.3	6.2	3.12	20.4	26.2	5.8	2.15	24.0	28.0	4.0
4YJ5	2.78	16.2	22.7	6.5	2.23	16.7	23.0	6.3	2.55	17.2	23.1	5.9	1.71	18.2	22.9	4.7
3L0W	3.28	29.7	31.4	1.7	2.84	23.4	27.0	3.6	3.06	23.0	26.7	3.7	2.05	25.5	27.2	1.7
6SZW	2.30	20.6	26.6	6.0	2.04	19.5	27.7	8.2	2.50	19.3	26.4	7.1	1.50	21.8	27.6	5.8
6KBI	2.68	23.0	27.4	4.4	2.71	22.0	28.0	6.0	2.72	21.7	26.9	5.2	2.24	22.9	27.3	4.4
8APY	2.28	27.2	32.8	5.6	2.63	25.9	33.6	7.7	2.02	26.5	32.3	5.8	1.50	28.4	33.1	4.7
8F0V	2.82	25.7	29.2	3.5	3.42	25.1	31.9	6.8	3.16	24.9	30.0	5.1	2.10	28.1	31.4	3.3
8HUK	2.49	23.0	26.7	3.7	2.92	20.8	28.7	7.9	2.32	21.1	26.9	5.8	1.72	23.0	26.4	3.4
1R30	3.80	20.6	24.6	4.0	2.53	18.4	24.5	6.1	3.47	18.0	23.9	5.9	2.13	21.1	25.1	4.0
1YNS	2.82	21.0	21.5	0.5	2.00	20.2	23.8	3.6	2.50	18.1	21.4	3.3	2.07	19.8	24.6	4.8
3JWR	3.19	20.9	27.7	6.8	2.60	19.1	27.5	8.4	2.87	20.7	27.8	7.1	1.99	22.0	26.7	4.7
1FT2	2.68	21.8	25.9	4.1	2.32	19.4	26.2	6.8	2.68	20.4	26.1	5.7	1.70	22.1	25.5	3.4
1D8U	2.63	21.0	21.0	0.0	2.24	20.1	25.8	5.7	2.05	19.9	24.3	4.4	1.93	21.0	24.8	3.8
1M52	2.62	20.3	24.7	4.4	2.10	18.0	24.1	6.1	2.28	18.7	24.1	5.4	1.99	18.4	24.1	5.7
1ZOY	3.24	20.5	25.2	4.7	2.88	19.5	25.4	5.9	3.00	19.2	24.7	5.5	2.31	21.5	25.4	3.9
Mean	*2.85*	*22.3*	*26.2*	*4.0*	*2.50*	*20.6*	*26.8*	*6.2*	*2.67*	*20.6*	*25.9*	*5.3*	*1.91*	*22.4*	*26.5*	*4.1*

[a]Data used to generate plots shown in **Fig. 3**.

Table S7: MolProbity score (MS), R_{work}, R_{free}, and ΔR (R_{free}-R_{work}) calculated from *de novo* refinement of 10 crystal structures by PHENIX, REFMAC5, and KNexPHENIX using maps extracted from the PDB.

PDB code[a]	Starting Model[b]	PHENIX[c]				REFMAC5[c]				KNexPHENIX[c]			
		MS	R_{work}	R_{free}	ΔR	MS	R_{work}	R_{free}	ΔR	MS	R_{work}	R_{free}	ΔR
1YNS	1ZS9	1.09	26.4	28.5	2.1	1.66	26.5	28.0	1.5	0.94	26.9	28.6	1.7
3L0W	1PLQ	2.79	25.8	29.6	3.8	2.65	25.0	27.2	2.2	1.45	29.8	30.5	0.7
1Y28	1DRM	1.95	27.1	30.0	2.9	2.08	27.1	29.3	2.2	0.91	28.4	30.6	2.2
1FT2	1FT1	2.39	19.7	26.1	6.4	2.43	21.0	25.9	4.9	1.06	25.1	27.8	2.7
1D8U	2GNW	2.23	25.5	30.3	4.8	2.67	25.8	29.1	3.3	1.46	27.0	30.5	3.5
6SZW	1P32	2.15	25.8	32.2	6.4	1.94	26.6	31.0	4.4	0.97	30.2	32.0	1.8
6KBI	1M6B	2.82	23.2	28.5	5.3	3.04	23.1	27.3	4.2	1.51	26.2	29.1	2.9
8APY	6I6J	2.54	28.4	34.7	6.3	1.85	29.4	33.8	4.4	1.58	31.3	35.0	3.7
8F0V	7BKX	3.08	28.5	36.4	8.1	3.32	27.5	33.6	6.1	1.64	34.4	39.0	4.6
8HUK	3SP6	2.80	24.7	32.1	7.4	2.18	25.1	29.6	4.5	1.54	28.9	32.4	3.5
	Mean	*2.38*	*25.5*	*30.9*	*5.3*	*2.38*	*25.7*	*29.5*	*3.8*	*1.31*	*28.8*	*31.6*	*2.7*

[a]Deposition corresponding to the map used for refinement.
[b]Model used for molecular replacement (MR).
[c]Data used to generate plots shown in **Fig. 4**.

Table S8: Clashscores calculated from deposited crystal structures and *de novo* refinement using PHENIX and KNexPHENIX.

PDB model	Deposited		*De novo*	
	PHENIX	KNexPHENIX	PHENIX	KNexPHENIX
1Y28	6.20	3.60	5.40	1.60
3UZ0	13.1	8.60	-	-
4YJ5	8.40	5.90	-	-
3L0W	13.6	9.20	10.6	4.40
6SZW	9.50	3.50	13.5	1.80
6KBI	13.7	8.90	13.0	3.80
8APY	14.1	7.90	11.1	4.20
8F0V	55.0	10.2	29.0	8.00
8HUK	15.6	8.80	16.8	10.4
1R30	28.6	15.4	-	-
1YNS	23.3	27.3	1.80	1.80
3JWR	11.6	9.30	-	-
1FT2	18.0	7.90	19.9	2.70
1D8U	9.70	7.30	7.90	3.50
1M52	8.40	6.30	-	-
1ZOY	20.8	12.1	-	-
Mean	*16.8*	*9.50*	*12.9*	*4.20*

Table S9: Clashscores calculated from deposited cryo-EM structures and *de novo* refinement using PHENIX and KNexPHENIX.

PDB model	Deposited		*De novo*	
	PHENIX	KNexPHENIX	PHENIX	KNexPHENIX
5AN9	11.7	6.10	14.3	6.70
8ETH	11.4	5.40	14.1	6.20
6OF4	7.60	2.50	8.30	1.40
7UN3	13.6	6.20	-	-
8ASW	18.2	6.10	-	-
7W0L	12.0	6.80	-	-
5A1A	12.9	5.90	8.60	3.10
8GUB	33.4	4.70	10.3	2.30
6JO5	15.3	9.20	4.50	0.90
6YEZ	14.9	12.2	5.80	1.60
5H1S	15.0	11.3	13.9	7.60
8GUD	16.1	5.90	14.3	2.00
7W0P	13.0	5.40	-	-
9E0N	-	-	17.2	8.50
Mean	*15.0*	*6.70*	*11.1*	*4.00*

Supplementary Methods

Default PHENIX refinement for cryo-EM and X-ray crystal structures

For performance comparisons in refinement of cryo-EM structures with KNexPHENIX, five cycles of “default” PHENIX refinement (*phenix.real_space_refine*) were performed using local grid search, global minimization, occupancy, N/Q/H flips, and B-factor refinement, with target root mean square deviation (RMSD) of 0.01Å and 1.0˚ for bonds and angles, respectively. Restraints such as secondary structure and Ramachandran were applied but not reference model restraints. For *pdb_interpretation*, Ramachandran restraints for only peptide bonds were added, but peptide planarity constraints were not employed. The dihedral function type was set to be determined by the sign of periodicity. Similarly, unless stated otherwise, for X-ray crystallographic structure refinement comparisons, five cycles of PHENIX refinement (*phenix.refine*) were performed using real-space, reciprocal-space, occupancy, and B-factor refinement. For *pdb_interpretation*, the dihedral function type was set to be determined by the sign of periodicity. For all the steps, parameters not described above were set to their default values. All the parameters in the two refinement procedures described here employed default values for parameters not described here.

Model refinement in REFMAC

Cryo-EM structures were refined using twenty cycles of REFMAC Servalcat (version 1.6.0) masked refinement with the weight and symmetry chosen automatically, and addition of hydrogen atoms. No RNA/DNA restraints were added, sharpening was not performed, and jellybody refinement was turned off. Crystal structures were refined in REFMAC5 (version 5.8.0419) through ten cycles of maximum likelihood restrained refinement using default parameters, e.g., isotropic B-factors, automatically optimized and experimental sigma stereochemistry/X-ray weights, and hydrogen atom addition. MolProbity scores[1], CC_{mask}[2], and R_{work}/R_{free}[3] values for the REFMAC-refined cryo-EM and crystal structures were calculated using the PHENIX validation tool. The MolProbity scores, R_{work}, and R_{free} values for the crystal structures deposited in PDB were also calculated similarly. Any options not explicitly mentioned were kept at their default values throughout.

Supplementary Results

Motivation for stage and parameter selection in the KNexPHENIX refinement pipeline

Selection of the stages and parameters in KNexPHENIX refinement was based on a defined rationale, although certain parameters were optimized empirically through trial and error. The guiding principle of the refinement protocol is to maximize the model quality while maintaining an appropriate map-to-model fit. The first step of the refinement pipeline involves the addition of H-atoms, which was suggested to improve the model geometry for crystal structures by correcting Asn/Gln/His flips and reducing clashes[1,4,5]. For the *de novo* refinement of crystal and cryo-EM structures, the next step involved refinement with simulated annealing, among other strategies, which improves agreement with the experimental map. Additionally, restraints on secondary structure, stereochemistry, reference model, etc., were applied to maintain chemically reasonable geometry. Although the PHENIX refinement of deposited cryo-EM models was observed to enhance the final KNexPHENIX refined model, we omitted this step for the crystal structures, as it did not appreciably alter the quality of the structure.

The subsequent step in the pipeline is geometry minimization, which is known to improve stereochemical parameters[6]. The number of minimization cycles was altered depending on (i) the confidence in the starting model quality, and (ii) eventual map-to-model fit. Deposited structures were subjected to a lower number of cycles (1-2) due to their extensive prior refinement and manual model corrections, thereby requiring modest improvements to improve their suitability for PDB deposition. In contrast, *de novo* models were passed

through multiple rounds of minimization (2-5 cycles) to improve the model quality. However, since the minimization process is independent of the model and therefore carries a risk of overcorrection, it was followed by an additional round of PHENIX refinement focusing on fitting the model to the map. This refinement was accompanied by several restraints, particularly the harmonic restraints on the starting coordinates, in addition to the ones present in the first PHENIX refinement, to prevent excessive deviations from the improved geometry.

All in all, the sequence of steps in KNexPHENIX is designed to enhance model quality while maintaining consistency with the experimental data, thereby producing a refined structure suitable for immediate deposition.

Typical duration and efficiency of KNexPHENIX refinement

The total runtime for the KNexPHENIX workflow depends on the molecular weight of the structure, with larger molecules requiring a longer time. To provide an estimate of the time required, we report the wall-clock time for the KNexPHENIX refinements (*de novo* and deposited) for two representative structures spanning the lowest and highest molecular weights obtained from cryo-EM and X-ray crystallography, respectively. The *de novo* refinement of crystal structures with PDB codes 1Y28 (15 kDa) and 6KBI (140 kDa) required 32.6 and 245 min, respectively. Similarly, the time required for re-refining deposited structures 1Y28 and 4YJ5 (229 kDa) was 11.3 and 72 min, respectively. For the cryo-EM structures 8GUD (15 kDa) and 8ETH (2,428 kDa), the *de novo* refinement takes 88 and 764 min, respectively, and 11.3 and 72 min, respectively, for re-refining the deposited structure.

The ReadySet step does not report the wall-clock time, and therefore, it was excluded from these estimates. Additionally, for the *de novo* refinement of the cryo-EM structures, the initial docking of the model into the map was performed manually and therefore could not be timed. Similarly, the removal of the H-atoms was also conducted manually via the command line interface and was not included in the timing analysis.

With respect to human intervention, steps such as PHASER, ReadySet, PHENIX refinement, and geometry minimization require manual input of the model or both the map and the model. Also, as noted above, docking of *de novo* models into cryo-EM maps and removal of H-atoms are manual processes. In contrast, default PHENIX typically requires the user to provide the model and map only once.

Regarding the efficiency of the KNexPHENIX protocol compared to the default approach, the latter often requires a substantial amount of time to manually correct the geometric outliers of the model. The time required for that is highly speculative, as it depends on user experience as well as the structural complexity. In contrast, KNexPHENIX requires minimal manual intervention in correcting geometric outliers while ensuring map-to-model fit and therefore has the potential to save time ranging from several days to several months.

Analyses of the effect of variation of stages and parameters in KNexPHENIX

The different steps and specific criteria in KNexPHENIX were varied in the *de novo* refinement of two crystal and cryo-EM structures (modified KNexPHENIX is referred to as KNexPHENIX-M) to highlight their importance. The structures were chosen to represent the range in size (low and high molecular weight), resolution (poor and better), and type of molecule (single molecule *vs.* complex). The MolProbity score (MS) and map-to-model fit parameters for specific parameter variations are listed below.

1. ***No reference model restraints (RMR) in the final PHENIX refinement for cryo-EM structures.*** These analyses used 6OF4 (3.20 Å, 227 kDa, protein), 8GUD (2.60 Å, 128 kDa, protein), and 5AN9 (3.30 Å, 1,419 kDa, nucleoprotein complex). Additionally, the reference coordinate restraints (RCR) were also removed in *pdb_interpretation.* Absence of the restraints affected the MolProbity score as the improvements in the model from the geometry minimization in the previous step were partially nullified.

PDB model[a]	Starting model[b]	KNexPHENIX		KNexPHENIX-M		
		MS	CC_{mask}	Modification	MS	CC_{mask}
6OF4	6OF2	1.29	0.74	No RMR	1.42	0.76
				No RMR+RCR	1.23	0.45
8GUD	8GUA	1.17	0.45	No RMR	2.17	0.77
				No RMR+RCR	2.13	0.50
5AN9	6QKL	1.76	0.80	No RMR	2.01	0.80
				No RMR+RCR	2.77	0.82

[a]Deposition corresponding to the map used for refinement.
[b]Model used for molecular replacement (MR).

2. ***Absence of simulated annealing (SA) in the final PHENIX refinement of crystal structures.*** These analyses used 1Y28 (2.10 Å, 15 kDa, protein), 8F0V (2.95 Å, 19 kDa, protein), and 6KBI (3.00 Å, 140 kDa, protein). As an alternative parameter variation, geometry restraints (GR) were also removed in the final step. In 1Y28, no SA reduced the map-to-model fit and thereby increased the R_{work} and R_{free}. On the other hand, removal of GR reduces the MolProbity score by affecting model quality. Interestingly, absence of SA or GR only affects the MolProbity score in 8F0V and 6KBI, reasons for which are not clearly understood.

PDB model[a]	Starting model[b]	KNexPHENIX				KNexPHENIX-M				
		MS	R_{work}	R_{free}	ΔR[c]	Modification	MS	R_{work}	R_{free}	ΔR[c]
1Y28	1DRM	0.91	28.4	30.6	2.20	No SA	0.91	29.3	31.5	2.20
						No GR	1.06	28.3	30.6	2.30
8F0V	7BKX	1.64	34.4	39	4.60	No SA	1.82	34.5	38.3	3.80
						No GR	1.97	31.1	36.7	5.60
6KBI	1M6B	1.51	26.2	29.1	2.90	No SA	1.84	26.2	29.3	3.10
						No GR	1.68	24.8	28.6	3.80

[a]Deposition corresponding to the map used for refinement. [b]Model used for molecular replacement (MR).
[c]ΔR is R_{free}-R_{work}

3. ***<u>Omission of final PHENIX refinement for the cryo-EM structures</u>***. Absence of the refinement affects both model quality and agreement with the map highlighting the importance of the refinement strategies and geometry restraints in this step.

PDB model[a]	Starting model[b]	KNexPHENIX		KNexPHENIX-M	
		MS	CC_{mask}	MS	CC_{mask}
6OF4	6OF2	1.29	0.74	1.43	0.72
8GUD	8GUA	1.17	0.45	1.28	0.43
5AN9	6QKL	1.76	0.80	1.83	0.77

[a]Deposition corresponding to the map used for refinement.
[b]Model used for molecular replacement (MR).

4. ***Removal of geometry minimization from the KNexPHENIX pipeline for crystal structures.*** As expected, failure to minimize the structure led to a considerable increase in the MolProbity scores.

PDB model[a]	Starting model[b]	KNexPHENIX				KNexPHENIX-M			
		MS	R_{work}	R_{free}	ΔR[c]	MS	R_{work}	R_{free}	ΔR[c]
1Y28	1DRM	0.91	28.4	30.6	2.20	1.58	26.9	29.6	2.70
8F0V	7BKX	1.64	34.4	39.0	4.60	2.74	31.7	34.8	3.10
6KBI	1M6B	1.51	26.2	29.1	2.90	2.75	24.7	27.7	3.00

[a]Deposition corresponding to the map used for refinement. [b]Model used for molecular replacement (MR). [c]ΔR is R_{free}-R_{work}

In conclusion, the alterations in parameters and stages successfully demonstrate their importance in the KNexPHENIX pipeline to obtain a model suitable for deposition.

Supplementary References

1. Davis, I. W.; Leaver-Fay, A.; Chen, V. B.; Block, J. N.; Kapral, G. J.; Wang, X.; Murray, L. W.; Arendall, W. B., 3rd; Snoeyink, J.; Richardson, J. S.; Richardson, D. C., MolProbity: all-atom contacts and structure validation for proteins and nucleic acids. *Nucleic Acids Res* **2007,** *35* (Web Server issue), W375-83.
2. Afonine, P. V.; Klaholz, B. P.; Moriarty, N. W.; Poon, B. K.; Sobolev, O. V.; Terwilliger, T. C.; Adams, P. D.; Urzhumtsev, A., New tools for the analysis and validation of cryo-EM maps and atomic models. *Acta Crystallogr D Struct Biol* **2018,** *74* (Pt 9), 814-840.
3. Wang, J., Estimation of the quality of refined protein crystal structures. *Protein Sci* **2015,** *24* (5), 661-9.
4. Afonine, P. V.; Adams, P. D., On the contribution of hydrogen atoms to X--ray scattering. *Computational Crystallography Newsletter 2012* (3), 18-21.
5. Headd, J. J.; Immormino, R. M.; Keedy, D. A.; Emsley, P.; Richardson, D. C.; Richardson, J. S., Autofix for backward-fit sidechains: using MolProbity and real-space refinement to put misfits in their place. *J Struct Funct Genomics* **2009,** *10* (1), 83-93.
6. phenix.geometry_minimization: regularize model geometry. https://www.phenix-online.org/version_docs/dev-2486/reference/geometry_minimization.html (accessed 01/04).

Case Study 1: PI3Kalpha H1047R cryo-EM structure (PDB code 8GUB)

Changes in validation parameters during each stage of *de novo* refinement of PI3Kalpha H1047R variant cryo-EM structure using **KNexPHENIX Workflow 2**.

PHENIX refinement

Refinement and model	
Model resolution (FSC 0.143, unmasked), Å	2.7
CC_{mask}	0.67
RMS deviations	
Bond lengths, Å	0.002
Bond angles, °	0.545
Validation	
MolProbity score	1.54
Clashscore	6.73
Rotamer outliers, %	0.00
Ramachandran plot (protein)	
Favored, %	97.0
Allowed, %	2.8
Disallowed, %	0.2

Geometry minimization

Refinement and model	
RMS deviations	
Bond lengths, Å	0.001
Bond angles, °	0.322
Validation	
Clashscore	3.03
Rotamer outliers, %	0.26
Ramachandran plot (protein)	
Favored, %	97.6
Allowed, %	2.3
Disallowed, %	0.1

PHENIX refinement

Refinement and model	
Model resolution (FSC 0.143, unmasked), Å	2.7
CC_{mask}	0.67
RMS deviations	
Bond lengths, Å	0.002
Bond angles, °	0.426
Validation	
MolProbity score	1.08
Clashscore	2.26
Rotamer outliers, %	0.26
Ramachandran plot (protein)	
Favored, %	97.6
Allowed, %	2.3
Disallowed, %	0.8

Case study 2: **Monoubiquitinated PCNA X-ray crystal structure (PDB code 3L0W)**

Changes in validation parameters during each stage of *de novo* refinement of monoubiquitinated PCNA crystal structure using **KNexPHENIX Workflow 4**.

PHENIX refinement

Refinement and model	
R_{work} (%)	27.45
R_{free} (%)	28.17
R_{free}-R_{work} (%)	0.72
RMS deviations	
Bond lengths, Å	0.013
Bond angles, °	1.836
Validation	
MolProbity score	2.39
Clashscore	19.16
Rotamer outliers, %	1.72
Ramachandran plot (protein)	
Favored, %	93.4
Allowed, %	4.7
Disallowed, %	1.9

Geometry minimization

Refinement and model	
RMS deviations	
Bond lengths, Å	0.001
Bond angles, °	0.384
Validation	
Clashscore	2.70
Rotamer outliers, %	0.86
Ramachandran plot (protein)	
Favored, %	96.9
Allowed, %	3.1
Disallowed, %	0.0

PHENIX refinement

Refinement and model	
R_{work} (%)	29.76
R_{free} (%)	30.53
R_{free}-R_{work} (%)	0.77
RMS deviations	
Bond lengths, Å	0.010
Bond angles, °	1.168
Validation	
MolProbity score	1.45
Clashscore	4.42
Rotamer outliers, %	0.86
Ramachandran plot (protein)	
Favored, %	96.5
Allowed, %	3.5
Disallowed, %	0.0

KNexPHEX "How-to" guide (Workflows 1-4 with PHENIX screenshots)

Workflow 1: Refinement of deposited cryo-EM models

1. Add hydrogen atoms to model

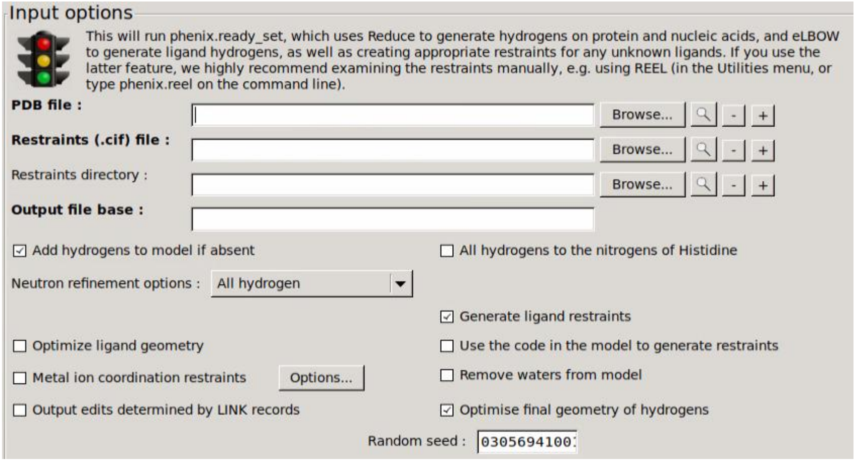

2. PHENIX refinement

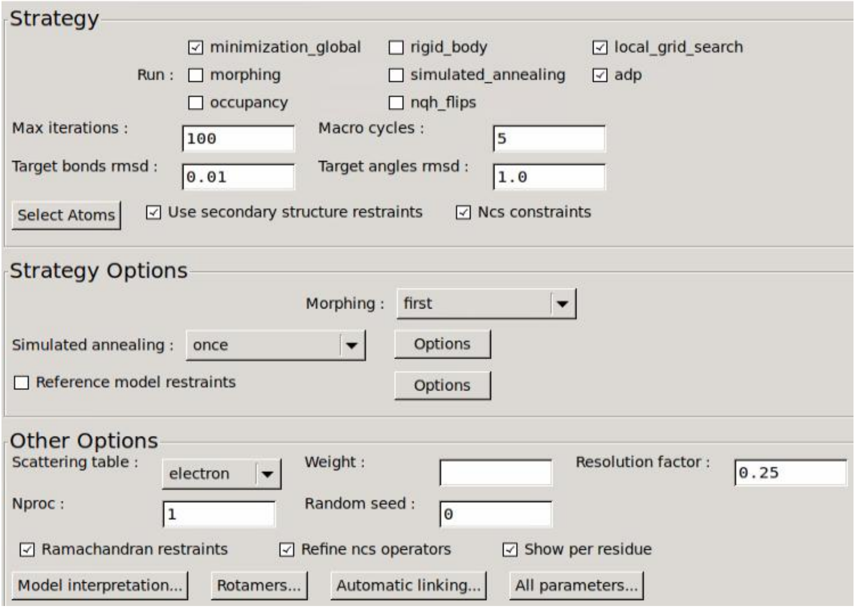

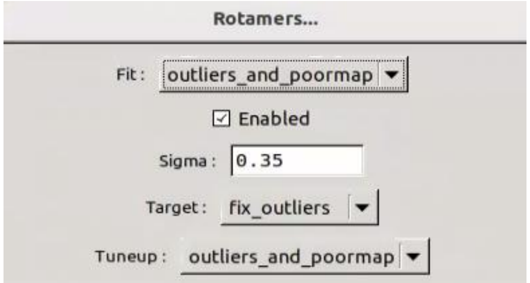

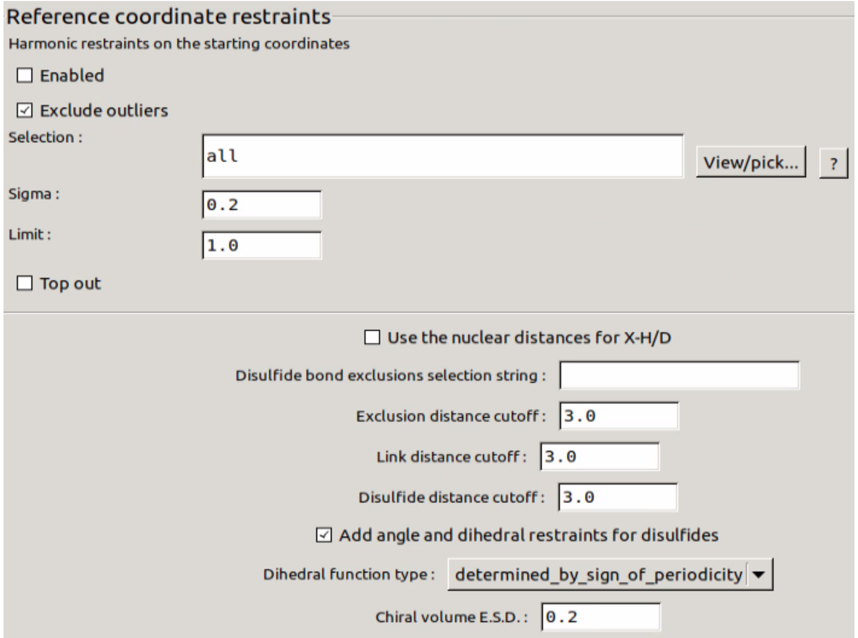
Reference coordinate restraints
Harmonic restraints on the starting coordinates
Enabled
Exclude outliers
Selection :
all
View/pick...
?
Sigma :
0.2
Limit :
1.0
Top out
Use the nuclear distances for X-H/D
Disulfide bond exclusions selection string :
Exclusion distance cutoff :
3.0
Link distance cutoff :
3.0
Disulfide distance cutoff :
3.0
Add angle and dihedral restraints for disulfides
Dihedral function type :
determined_by_sign_of_periodicity
Chiral volume E.S.D. :
0.2

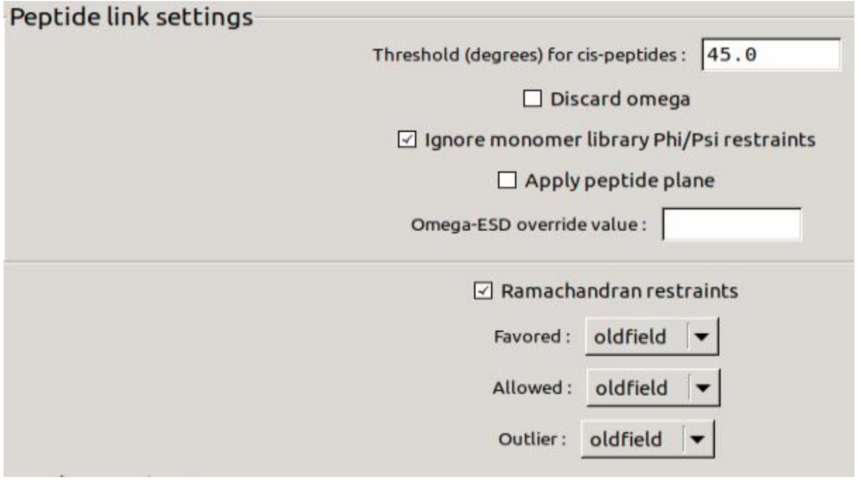
Peptide link settings
Threshold (degrees) for cis-peptides :
45.0
Discard omega
Ignore monomer library Phi/Psi restraints
Apply peptide plane
Omega-ESD override value :
Ramachandran restraints
Favored :
oldfield
Allowed :
oldfield
Outlier :
oldfield

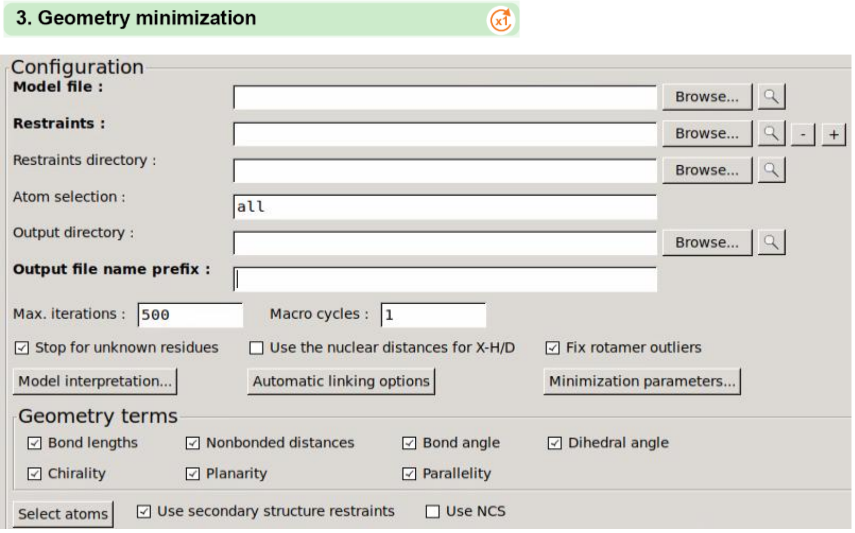
3. Geometry minimization
Configuration
Model file :
Browse...
Restraints :
Browse...
-
+
Restraints directory :
Browse...
Atom selection :
all
Output directory :
Browse...
Output file name prefix :
Max. iterations :
500
Macro cycles :
1
Stop for unknown residues
Use the nuclear distances for X-H/D
Fix rotamer outliers
Model interpretation...
Automatic linking options
Minimization parameters...
Geometry terms
Bond lengths
Nonbonded distances
Bond angle
Dihedral angle
Chirality
Planarity
Parallelity
Select atoms
Use secondary structure restraints
Use NCS

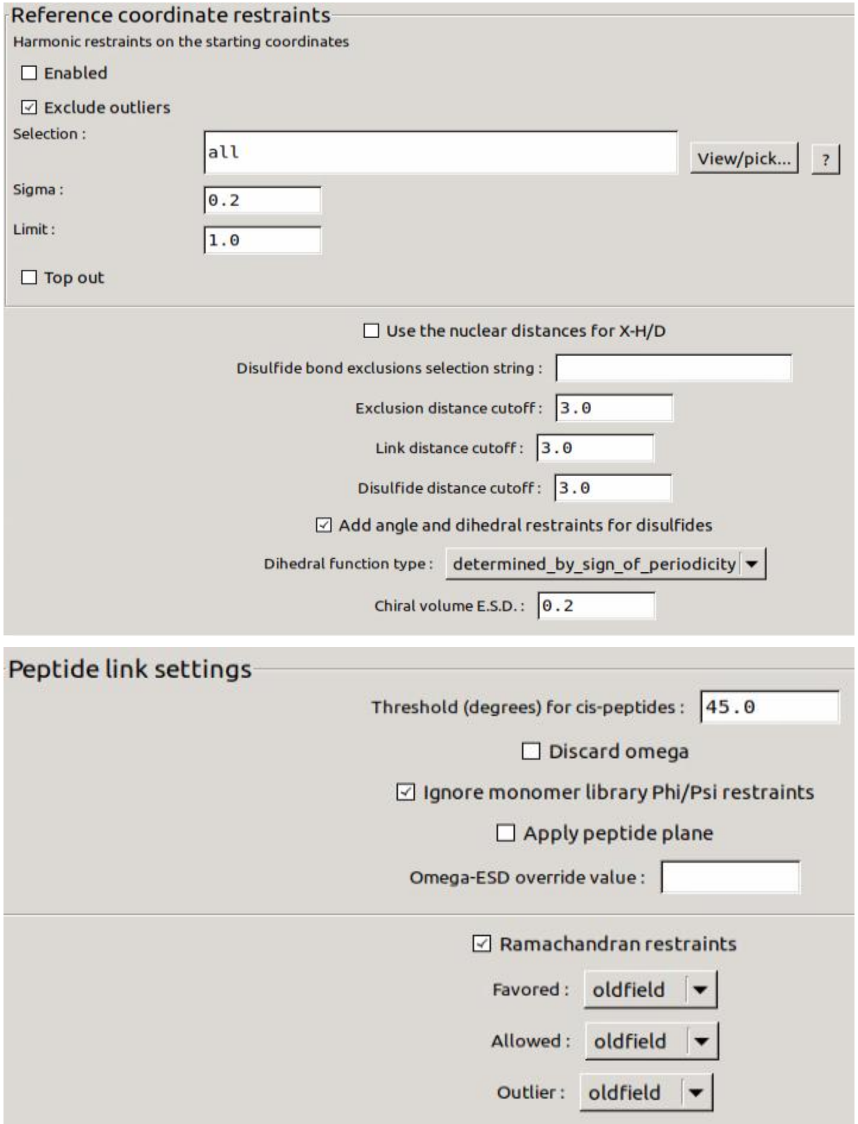

Reference coordinate restraints
Harmonic restraints on the starting coordinates
Enabled
Exclude outliers
Selection :
all
View/pick...
?
Sigma :
0.2
Limit :
1.0
Top out
Use the nuclear distances for X-H/D
Disulfide bond exclusions selection string :
Exclusion distance cutoff :
3.0
Link distance cutoff :
3.0
Disulfide distance cutoff :
3.0
Add angle and dihedral restraints for disulfides
Dihedral function type :
determined_by_sign_of_periodicity
Chiral volume E.S.D. :
0.2
Peptide link settings
Threshold (degrees) for cis-peptides :
45.0
Discard omega
Ignore monomer library Phi/Psi restraints
Apply peptide plane
Omega-ESD override value :
Ramachandran restraints
Favored :
oldfield
Allowed :
oldfield
Outlier :
oldfield

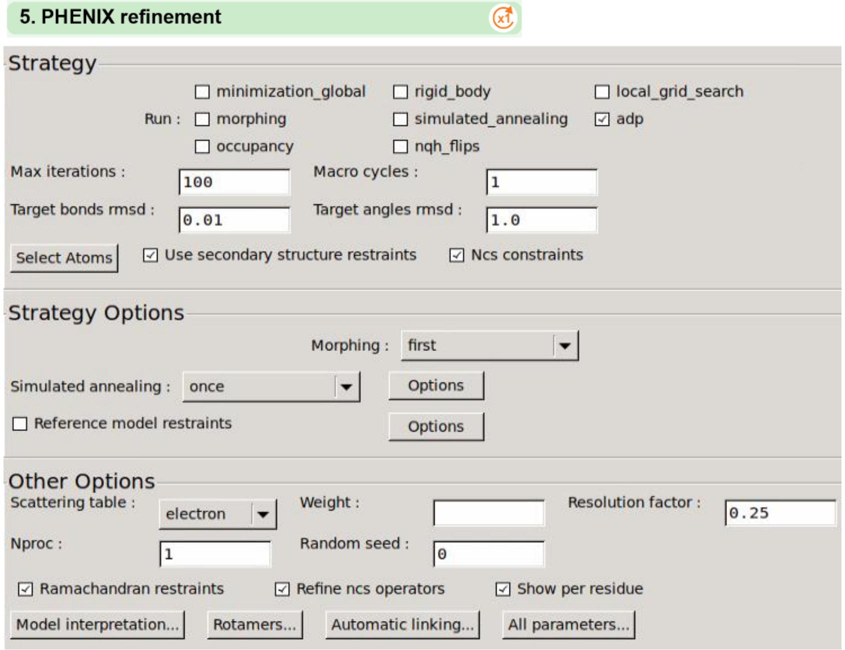

5. PHENIX refinement
Strategy
Run :
minimization_global
rigid_body
local_grid_search
morphing
simulated_annealing
adp
occupancy
nqh_flips
Max iterations :
100
Macro cycles :
1
Target bonds rmsd :
0.01
Target angles rmsd :
1.0
Select Atoms
Use secondary structure restraints
Ncs constraints
Strategy Options
Morphing :
first
Simulated annealing :
once
Options
Reference model restraints
Options
Other Options
Scattering table :
electron
Weight :
Resolution factor :
0.25
Nproc :
1
Random seed :
0
Ramachandran restraints
Refine ncs operators
Show per residue
Model interpretation...
Rotamers...
Automatic linking...
All parameters...

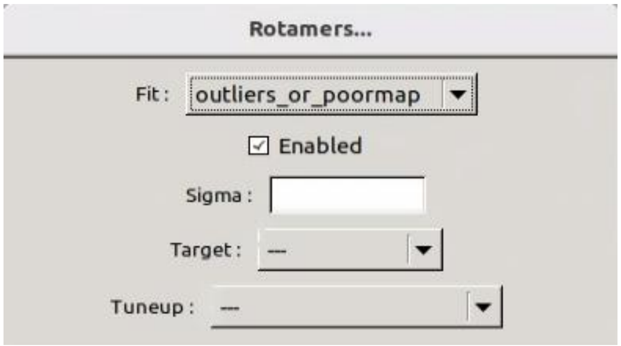
Rotamers...
Fit : outliers_or_poormap
Enabled
Sigma :
Target : ---
Tuneup : ---

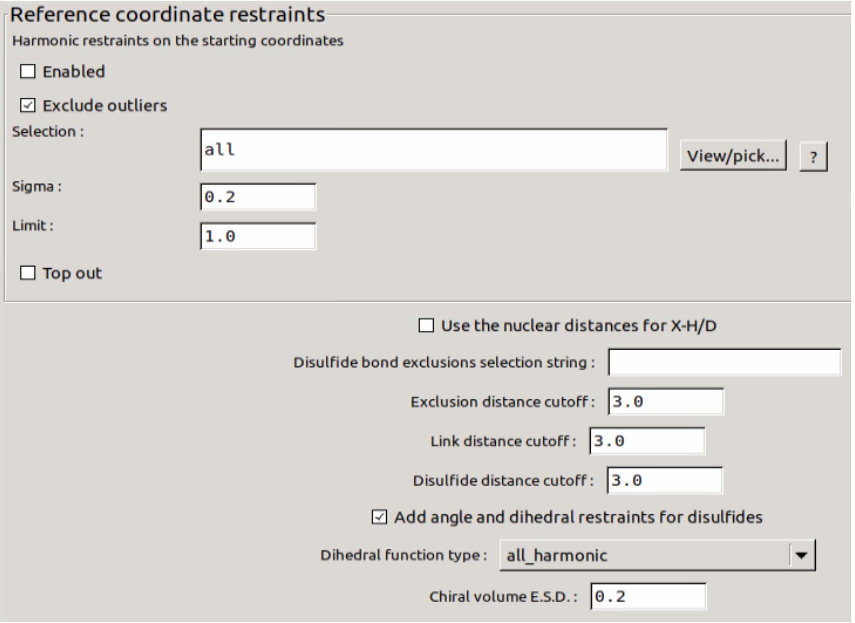
Reference coordinate restraints
Harmonic restraints on the starting coordinates
Enabled
Exclude outliers
Selection :
all
View/pick...
?
Sigma :
0.2
Limit :
1.0
Top out
Use the nuclear distances for X-H/D
Disulfide bond exclusions selection string :
Exclusion distance cutoff : 3.0
Link distance cutoff : 3.0
Disulfide distance cutoff : 3.0
Add angle and dihedral restraints for disulfides
Dihedral function type : all_harmonic
Chiral volume E.S.D. : 0.2

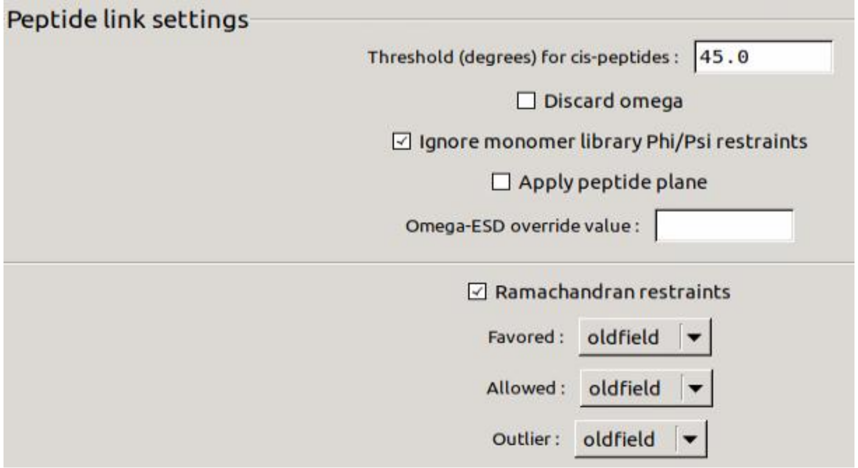
Peptide link settings
Threshold (degrees) for cis-peptides : 45.0
Discard omega
Ignore monomer library Phi/Psi restraints
Apply peptide plane
Omega-ESD override value :
Ramachandran restraints
Favored : oldfield
Allowed : oldfield
Outlier : oldfield

Workflow 2: Refinement of *de novo* cryo-EM models

2. Add hydrogen atoms to model

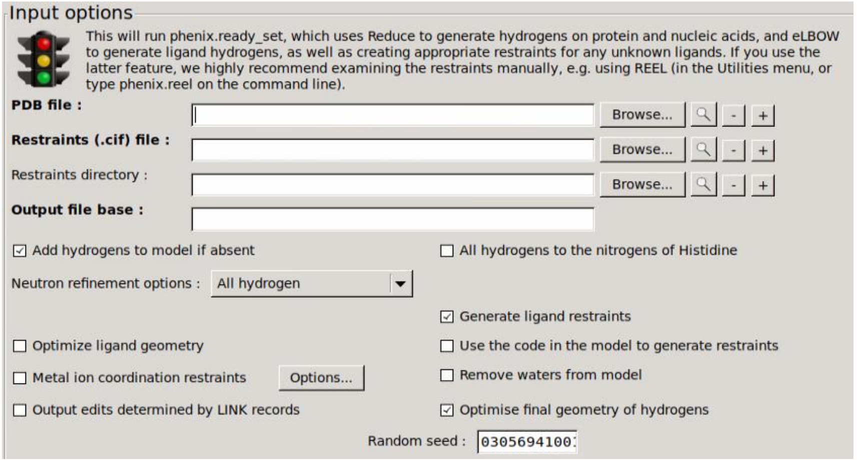

3. PHENIX refinement

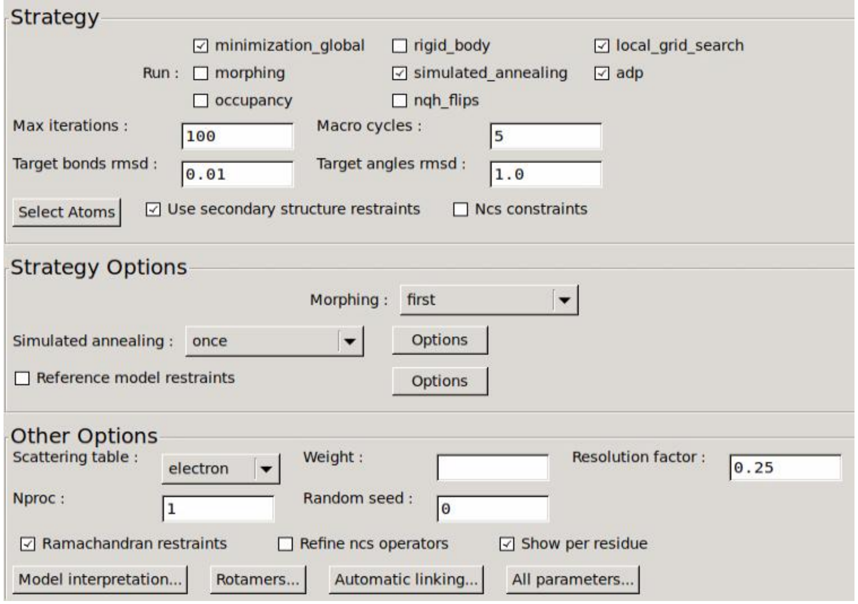

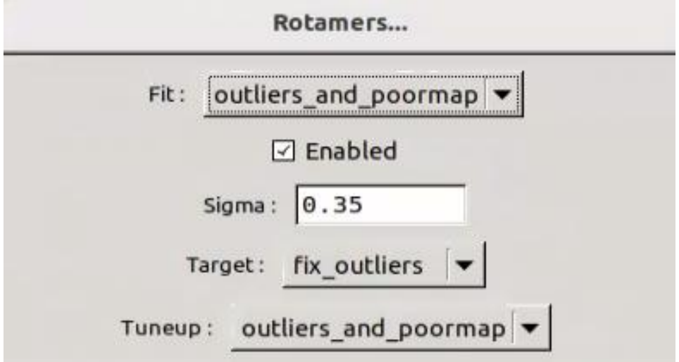

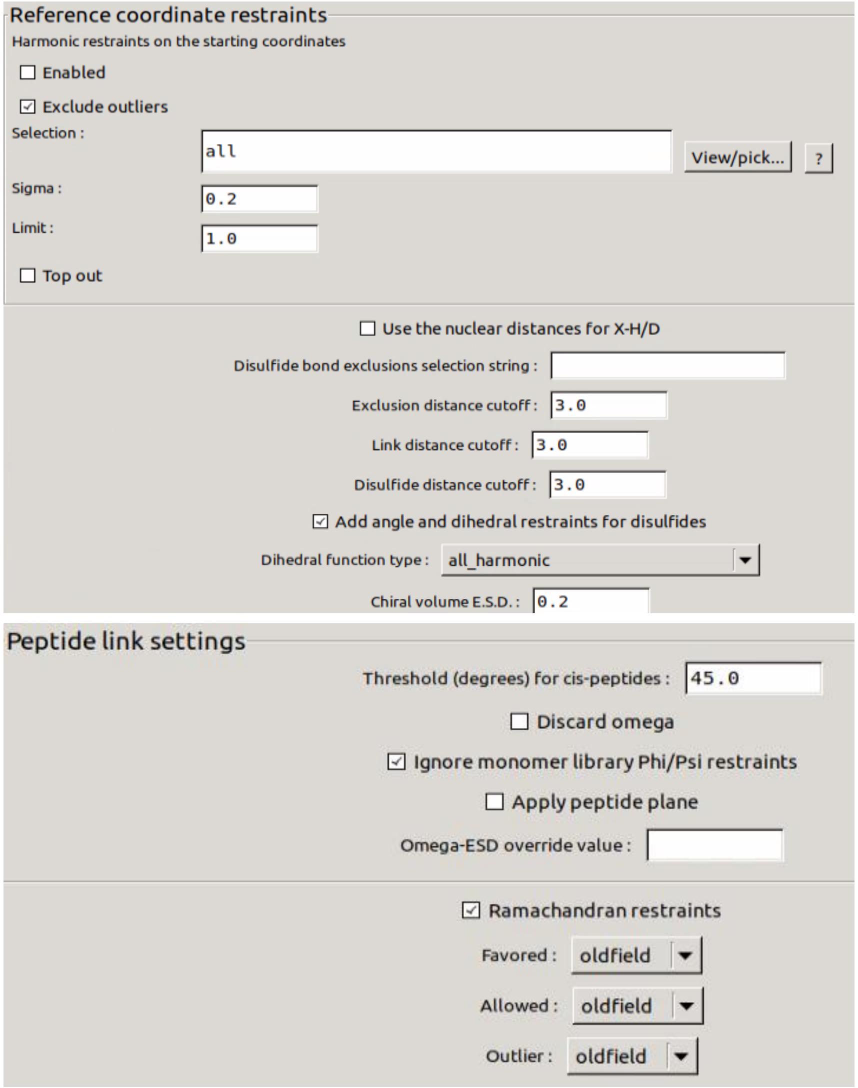
Reference coordinate restraints
Harmonic restraints on the starting coordinates
Enabled
Exclude outliers
Selection :
all
View/pick...
?
Sigma :
0.2
Limit :
1.0
Top out
Use the nuclear distances for X-H/D
Disulfide bond exclusions selection string :
Exclusion distance cutoff :
3.0
Link distance cutoff :
3.0
Disulfide distance cutoff :
3.0
Add angle and dihedral restraints for disulfides
Dihedral function type :
all_harmonic
Chiral volume E.S.D. :
0.2
Peptide link settings
Threshold (degrees) for cis-peptides :
45.0
Discard omega
Ignore monomer library Phi/Psi restraints
Apply peptide plane
Omega-ESD override value :
Ramachandran restraints
Favored :
oldfield
Allowed :
oldfield
Outlier :
oldfield

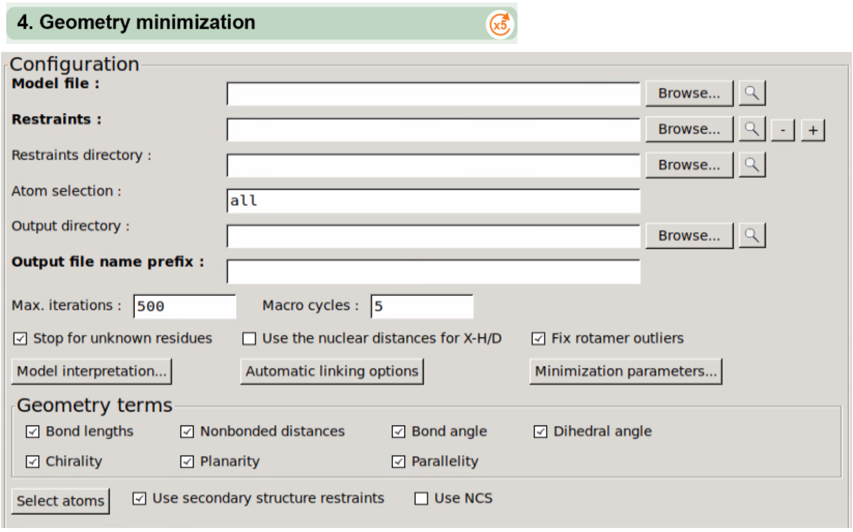
4. Geometry minimization
Configuration
Model file :
Browse...
Restraints :
Browse...
Restraints directory :
Browse...
Atom selection :
all
Output directory :
Browse...
Output file name prefix :
Max. iterations :
500
Macro cycles :
5
Stop for unknown residues
Use the nuclear distances for X-H/D
Fix rotamer outliers
Model interpretation...
Automatic linking options
Minimization parameters...
Geometry terms
Bond lengths
Nonbonded distances
Bond angle
Dihedral angle
Chirality
Planarity
Parallelity
Select atoms
Use secondary structure restraints
Use NCS

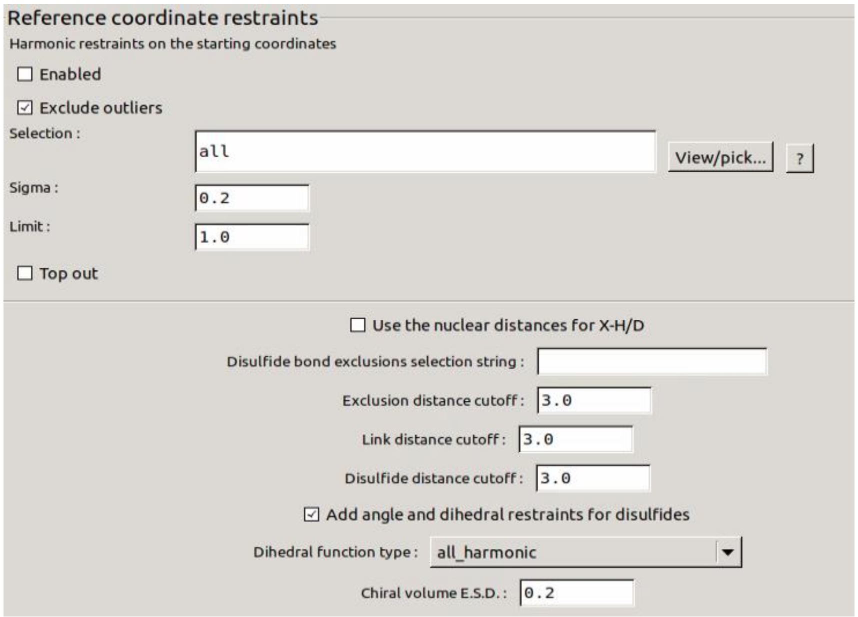
Reference coordinate restraints
Harmonic restraints on the starting coordinates
Enabled
Exclude outliers
Selection :
all
View/pick...
?
Sigma :
0.2
Limit :
1.0
Top out
Use the nuclear distances for X-H/D
Disulfide bond exclusions selection string :
Exclusion distance cutoff :
3.0
Link distance cutoff :
3.0
Disulfide distance cutoff :
3.0
Add angle and dihedral restraints for disulfides
Dihedral function type :
all_harmonic
Chiral volume E.S.D. :
0.2

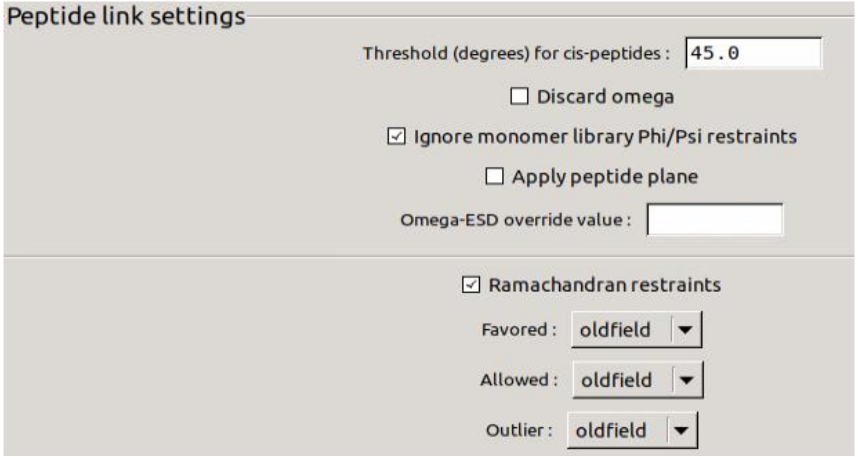
Peptide link settings
Threshold (degrees) for cis-peptides :
45.0
Discard omega
Ignore monomer library Phi/Psi restraints
Apply peptide plane
Omega-ESD override value :
Ramachandran restraints
Favored :
oldfield
Allowed :
oldfield
Outlier :
oldfield

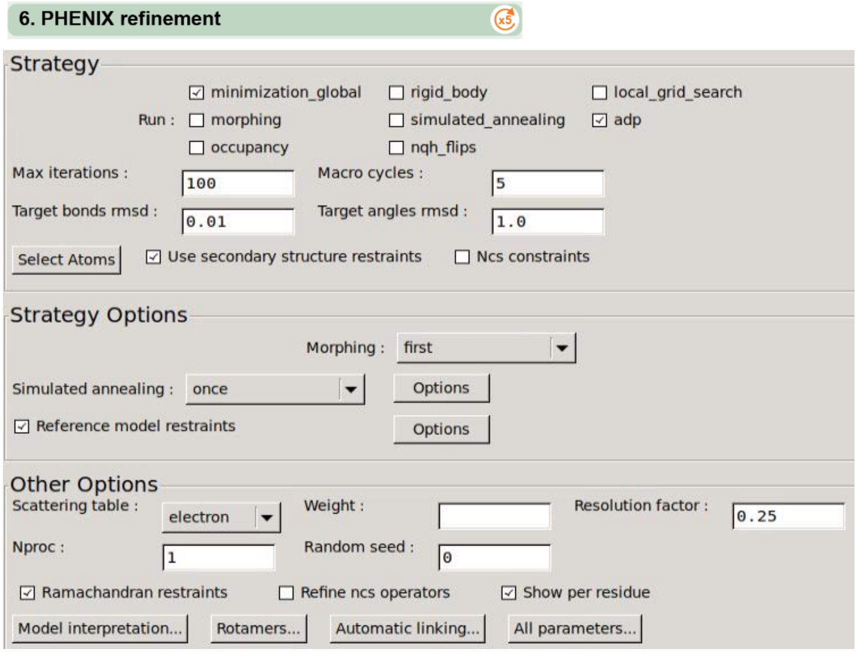
6. PHENIX refinement
Strategy
minimization_global
rigid_body
local_grid_search
Run :
morphing
simulated_annealing
adp
occupancy
nqh_flips
Max iterations :
100
Macro cycles :
5
Target bonds rmsd :
0.01
Target angles rmsd :
1.0
Select Atoms
Use secondary structure restraints
Ncs constraints
Strategy Options
Morphing :
first
Simulated annealing :
once
Options
Reference model restraints
Options
Other Options
Scattering table :
electron
Weight :
Resolution factor :
0.25
Nproc :
1
Random seed :
0
Ramachandran restraints
Refine ncs operators
Show per residue
Model interpretation...
Rotamers...
Automatic linking...
All parameters...

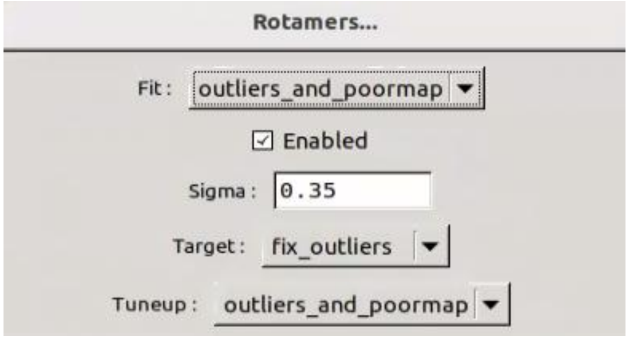
Rotamers...
Fit : outliers_and_poormap
Enabled
Sigma : 0.35
Target : fix_outliers
Tuneup : outliers_and_poormap

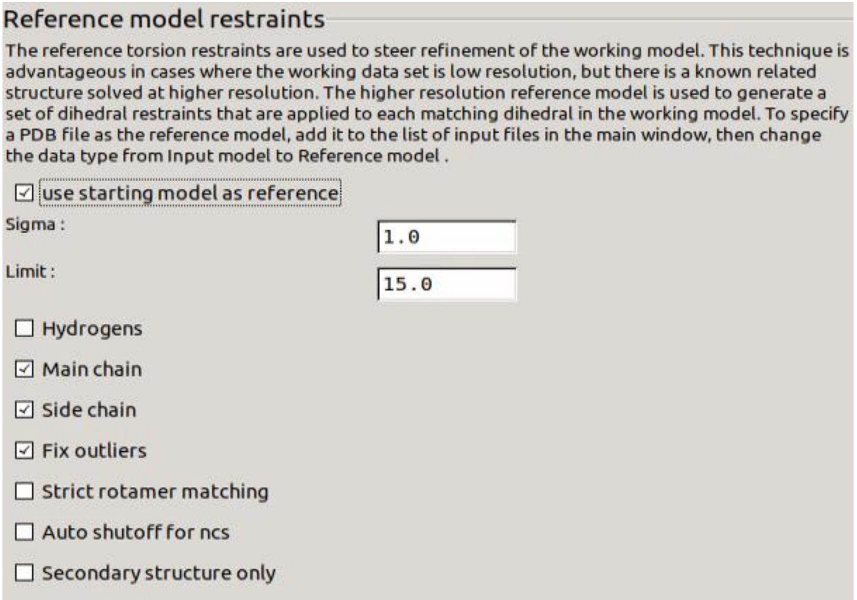
Reference model restraints
The reference torsion restraints are used to steer refinement of the working model. This technique is advantageous in cases where the working data set is low resolution, but there is a known related structure solved at higher resolution. The higher resolution reference model is used to generate a set of dihedral restraints that are applied to each matching dihedral in the working model. To specify a PDB file as the reference model, add it to the list of input files in the main window, then change the data type from Input model to Reference model .
use starting model as reference
Sigma :
1.0
Limit :
15.0
Hydrogens
Main chain
Side chain
Fix outliers
Strict rotamer matching
Auto shutoff for ncs
Secondary structure only

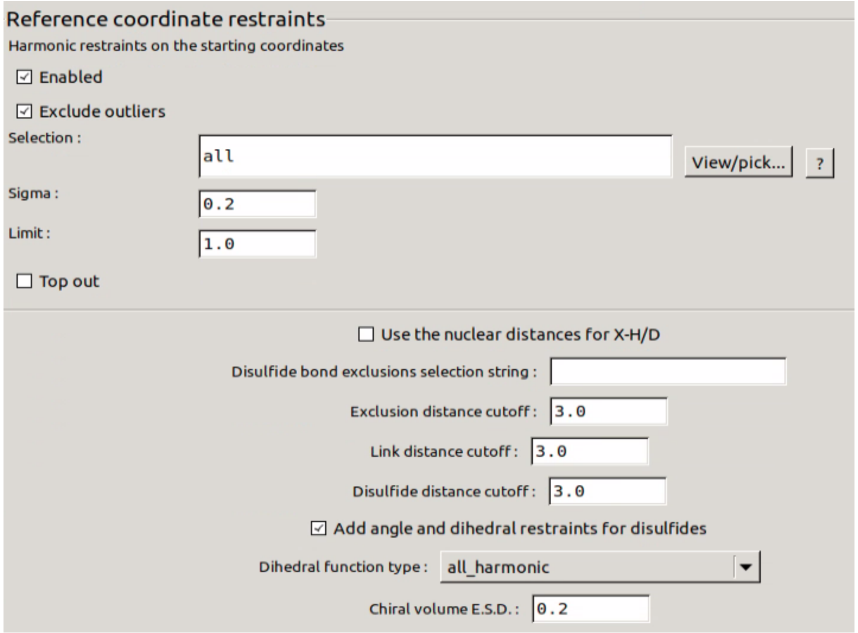
Reference coordinate restraints
Harmonic restraints on the starting coordinates
Enabled
Exclude outliers
Selection :
all
View/pick...
?
Sigma :
0.2
Limit :
1.0
Top out
Use the nuclear distances for X-H/D
Disulfide bond exclusions selection string :
Exclusion distance cutoff : 3.0
Link distance cutoff : 3.0
Disulfide distance cutoff : 3.0
Add angle and dihedral restraints for disulfides
Dihedral function type : all_harmonic
Chiral volume E.S.D. : 0.2

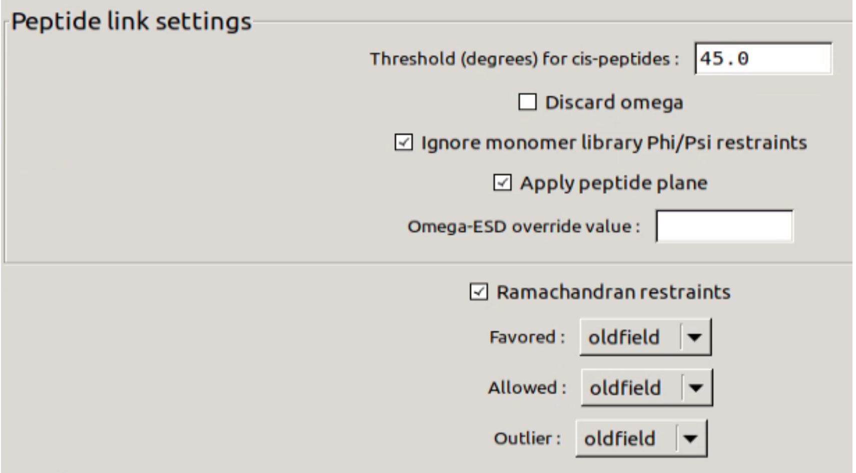
Peptide link settings
Threshold (degrees) for cis-peptides : 45.0
Discard omega
Ignore monomer library Phi/Psi restraints
Apply peptide plane
Omega-ESD override value :
Ramachandran restraints
Favored : oldfield
Allowed : oldfield
Outlier : oldfield

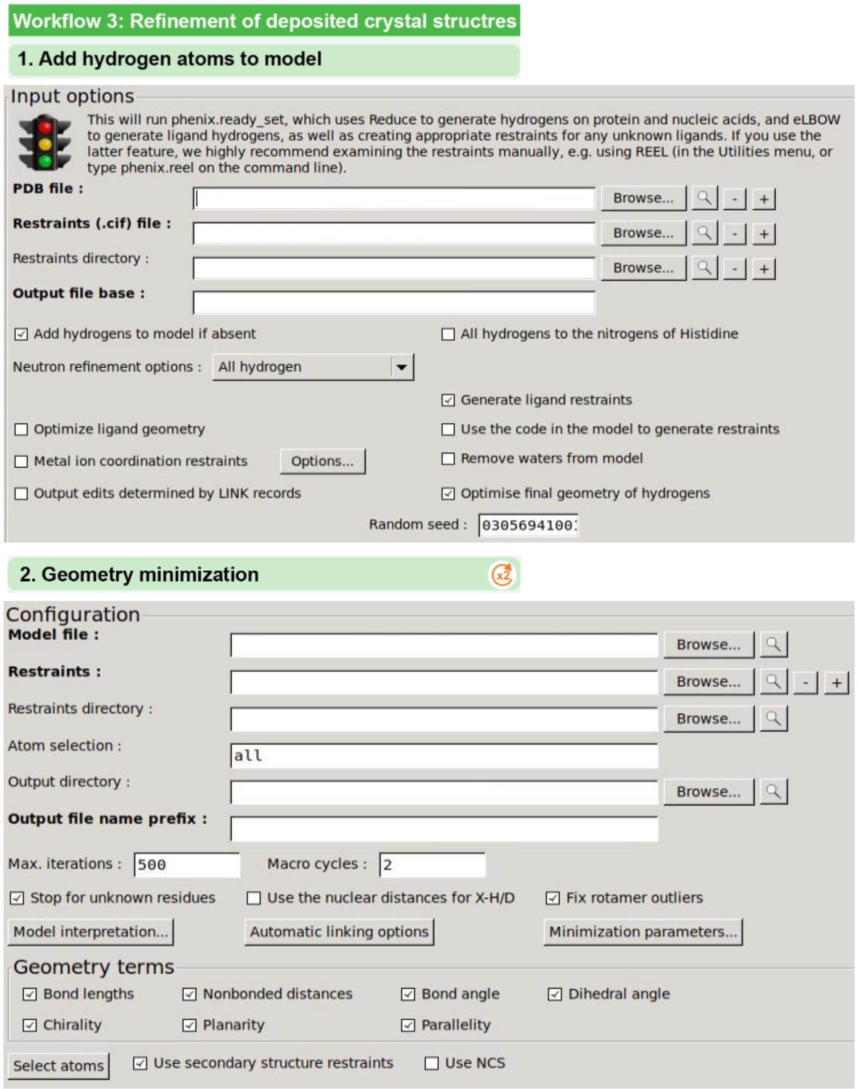
Workflow 3: Refinement of deposited crystal structres
1. Add hydrogen atoms to model
Input options
This will run phenix.ready_set, which uses Reduce to generate hydrogens on protein and nucleic acids, and eLBOW to generate ligand hydrogens, as well as creating appropriate restraints for any unknown ligands. If you use the latter feature, we highly recommend examining the restraints manually, e.g. using REEL (in the Utilities menu, or type phenix.reel on the command line).
PDB file :
Browse...
Restraints (.cif) file :
Browse...
Restraints directory :
Browse...
Output file base :
Add hydrogens to model if absent
All hydrogens to the nitrogens of Histidine
Neutron refinement options : All hydrogen
Generate ligand restraints
Optimize ligand geometry
Use the code in the model to generate restraints
Metal ion coordination restraints
Options...
Remove waters from model
Output edits determined by LINK records
Optimise final geometry of hydrogens
Random seed : 0305694100
2. Geometry minimization
Configuration
Model file :
Browse...
Restraints :
Browse...
Restraints directory :
Browse...
Atom selection : all
Output directory :
Browse...
Output file name prefix :
Max. iterations : 500
Macro cycles : 2
Stop for unknown residues
Use the nuclear distances for X-H/D
Fix rotamer outliers
Model interpretation...
Automatic linking options
Minimization parameters...
Geometry terms
Bond lengths
Nonbonded distances
Bond angle
Dihedral angle
Chirality
Planarity
Parallelity
Select atoms
Use secondary structure restraints
Use NCS

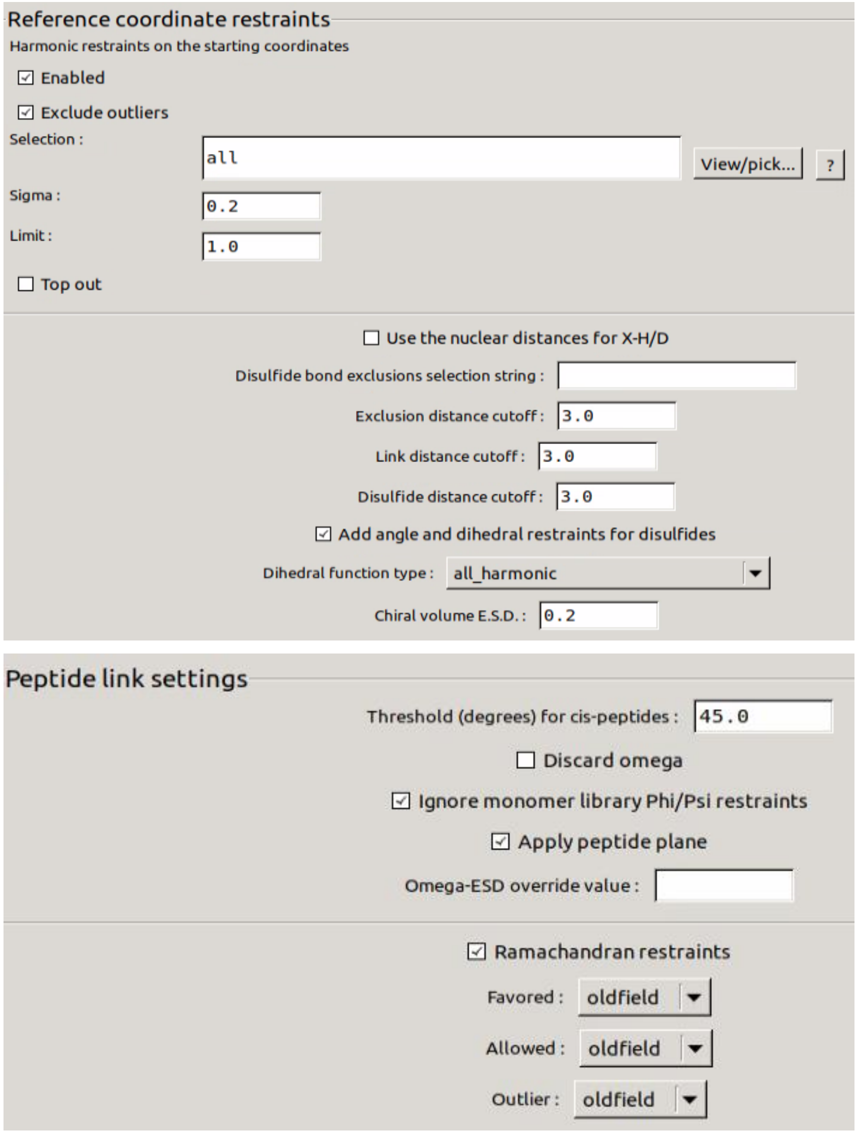
Reference coordinate restraints
Harmonic restraints on the starting coordinates
Enabled
Exclude outliers
Selection :
all
View/pick...
?
Sigma :
0.2
Limit :
1.0
Top out
Use the nuclear distances for X-H/D
Disulfide bond exclusions selection string :
Exclusion distance cutoff :
3.0
Link distance cutoff :
3.0
Disulfide distance cutoff :
3.0
Add angle and dihedral restraints for disulfides
Dihedral function type :
all_harmonic
Chiral volume E.S.D. :
0.2
Peptide link settings
Threshold (degrees) for cis-peptides :
45.0
Discard omega
Ignore monomer library Phi/Psi restraints
Apply peptide plane
Omega-ESD override value :
Ramachandran restraints
Favored :
oldfield
Allowed :
oldfield
Outlier :
oldfield

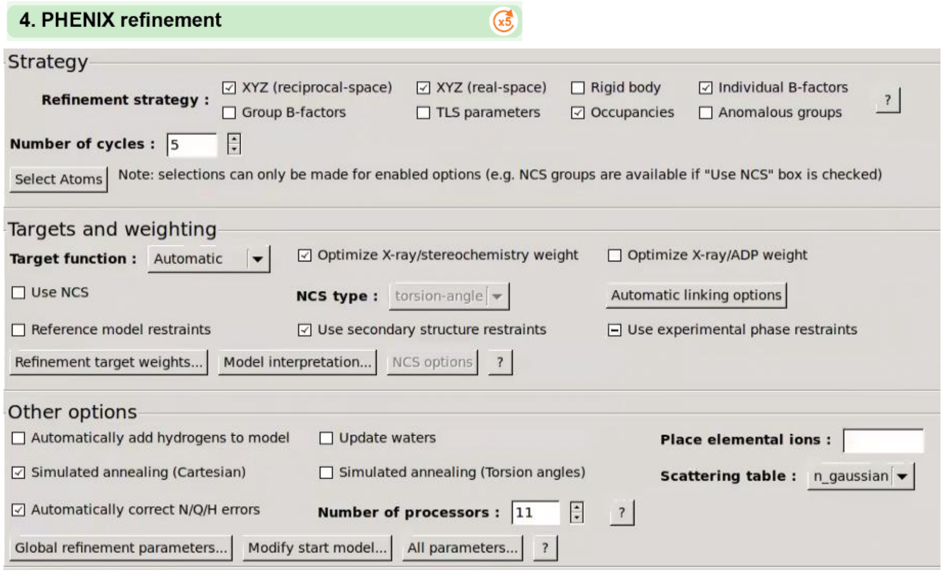
4. PHENIX refinement
Strategy
Refinement strategy :
XYZ (reciprocal-space)
XYZ (real-space)
Rigid body
Individual B-factors
Group B-factors
TLS parameters
Occupancies
Anomalous groups
Number of cycles : 5
Select Atoms
Note: selections can only be made for enabled options (e.g. NCS groups are available if "Use NCS" box is checked)
Targets and weighting
Target function : Automatic
Optimize X-ray/stereochemistry weight
Optimize X-ray/ADP weight
Use NCS
NCS type : torsion-angle
Automatic linking options
Reference model restraints
Use secondary structure restraints
Use experimental phase restraints
Refinement target weights...
Model interpretation...
NCS options
Other options
Automatically add hydrogens to model
Update waters
Place elemental ions :
Simulated annealing (Cartesian)
Simulated annealing (Torsion angles)
Scattering table : n_gaussian
Automatically correct N/Q/H errors
Number of processors : 11
Global refinement parameters...
Modify start model...
All parameters...

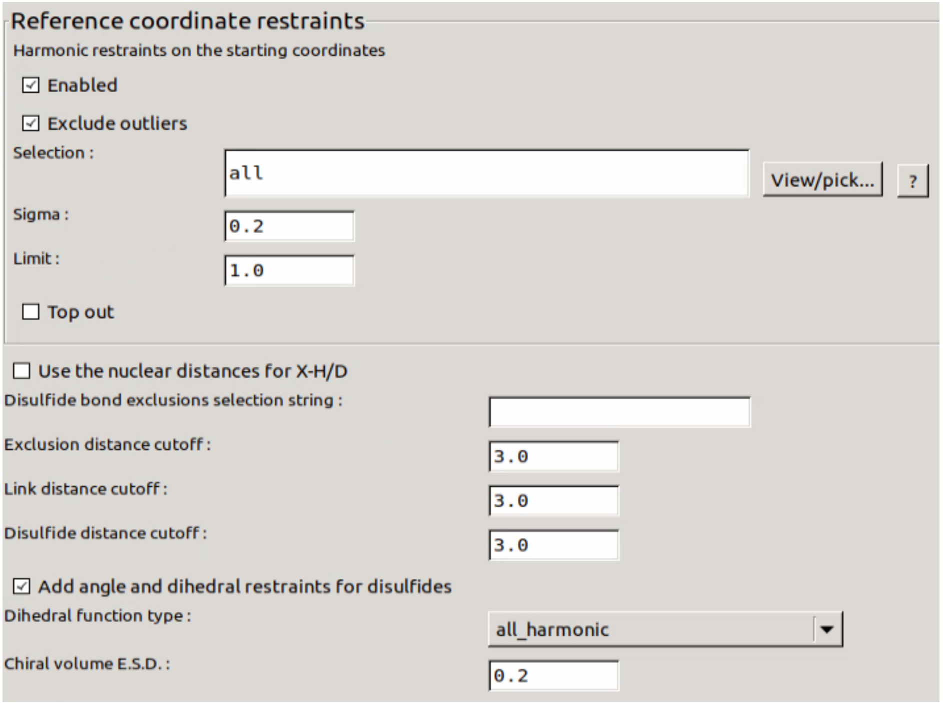
Reference coordinate restraints
Harmonic restraints on the starting coordinates
Enabled
Exclude outliers
Selection : all
View/pick...
Sigma : 0.2
Limit : 1.0
Top out
Use the nuclear distances for X-H/D
Disulfide bond exclusions selection string :
Exclusion distance cutoff : 3.0
Link distance cutoff : 3.0
Disulfide distance cutoff : 3.0
Add angle and dihedral restraints for disulfides
Dihedral function type : all_harmonic
Chiral volume E.S.D. : 0.2

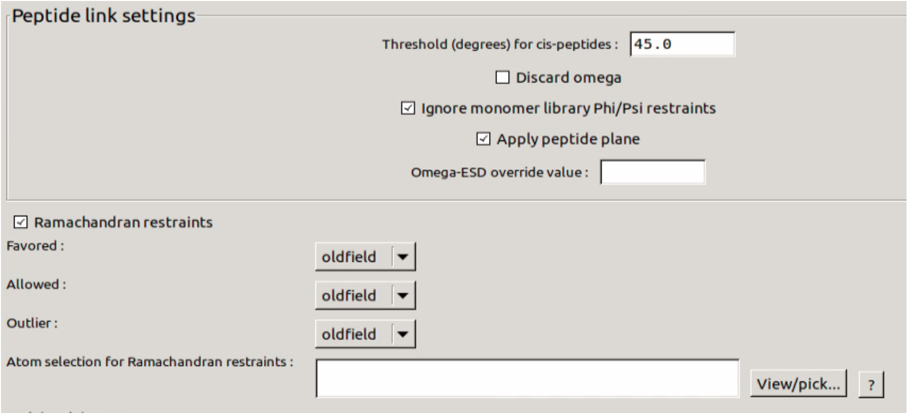
Peptide link settings
Threshold (degrees) for cis-peptides : 45.0
Discard omega
Ignore monomer library Phi/Psi restraints
Apply peptide plane
Omega-ESD override value :
Ramachandran restraints
Favored : oldfield
Allowed : oldfield
Outlier : oldfield
Atom selection for Ramachandran restraints :
View/pick...

Workflow 4: Refinement of *de novo* crystal structres

2. Add hydrogen atoms to model

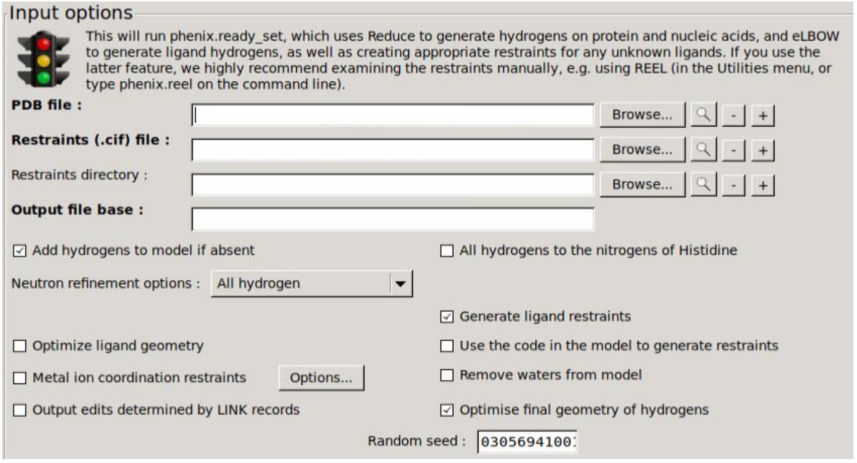

3. PHENIX refinement

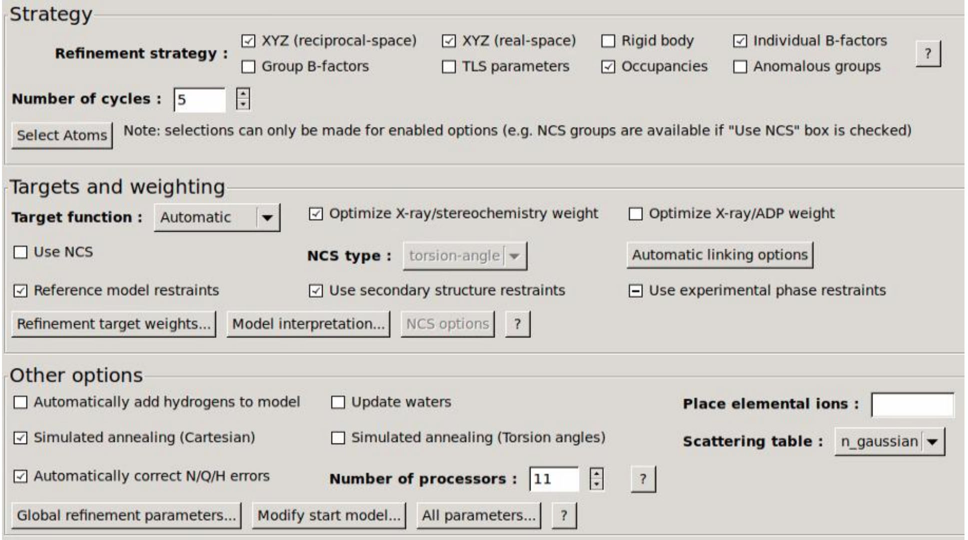

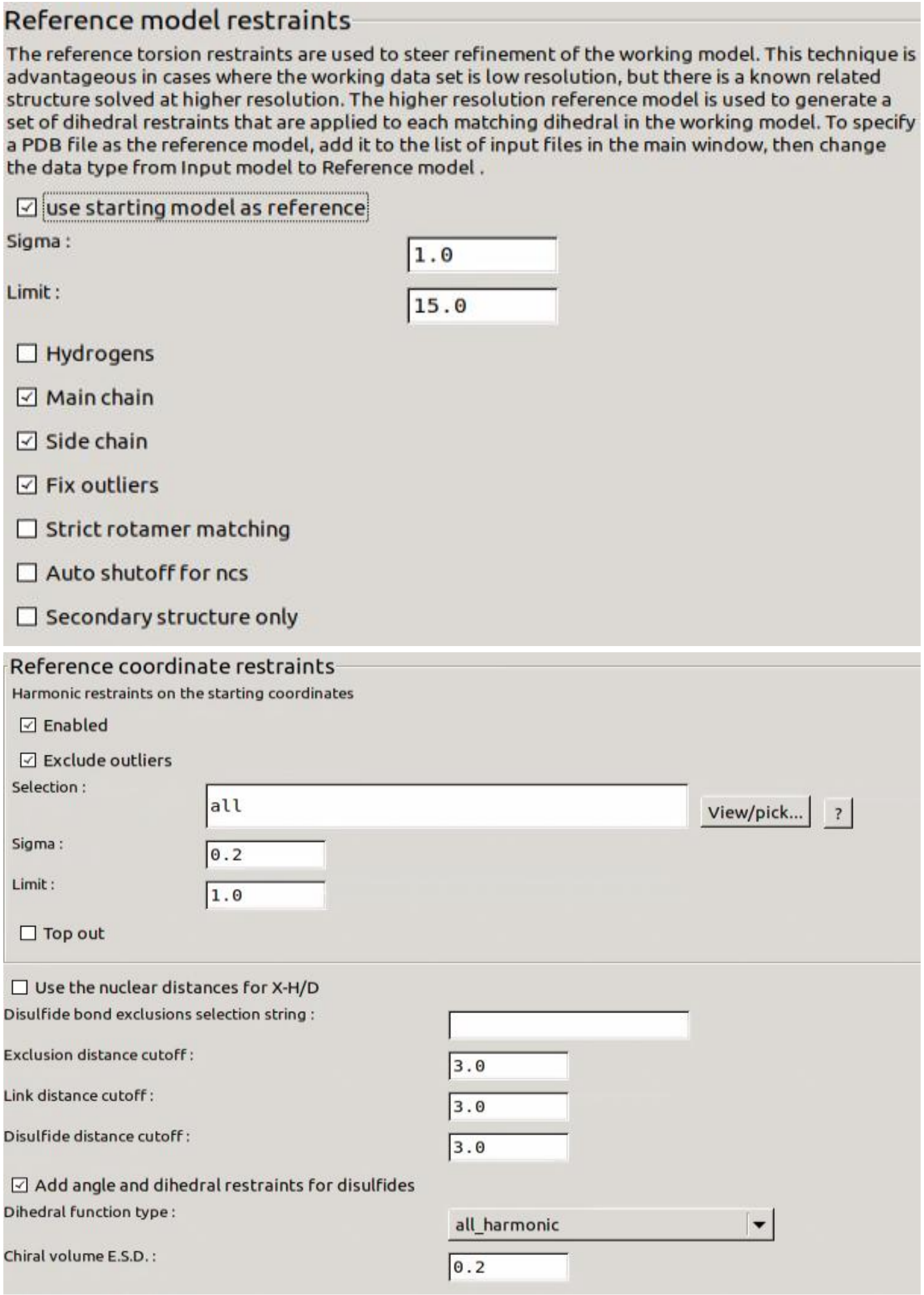
Reference model restraints
The reference torsion restraints are used to steer refinement of the working model. This technique is advantageous in cases where the working data set is low resolution, but there is a known related structure solved at higher resolution. The higher resolution reference model is used to generate a set of dihedral restraints that are applied to each matching dihedral in the working model. To specify a PDB file as the reference model, add it to the list of input files in the main window, then change the data type from Input model to Reference model .
use starting model as reference
Sigma :
1.0
Limit :
15.0
Hydrogens
Main chain
Side chain
Fix outliers
Strict rotamer matching
Auto shutoff for ncs
Secondary structure only
Reference coordinate restraints
Harmonic restraints on the starting coordinates
Enabled
Exclude outliers
Selection :
all
View/pick...
?
Sigma :
0.2
Limit :
1.0
Top out
Use the nuclear distances for X-H/D
Disulfide bond exclusions selection string :
Exclusion distance cutoff :
3.0
Link distance cutoff :
3.0
Disulfide distance cutoff :
3.0
Add angle and dihedral restraints for disulfides
Dihedral function type :
all_harmonic
Chiral volume E.S.D. :
0.2

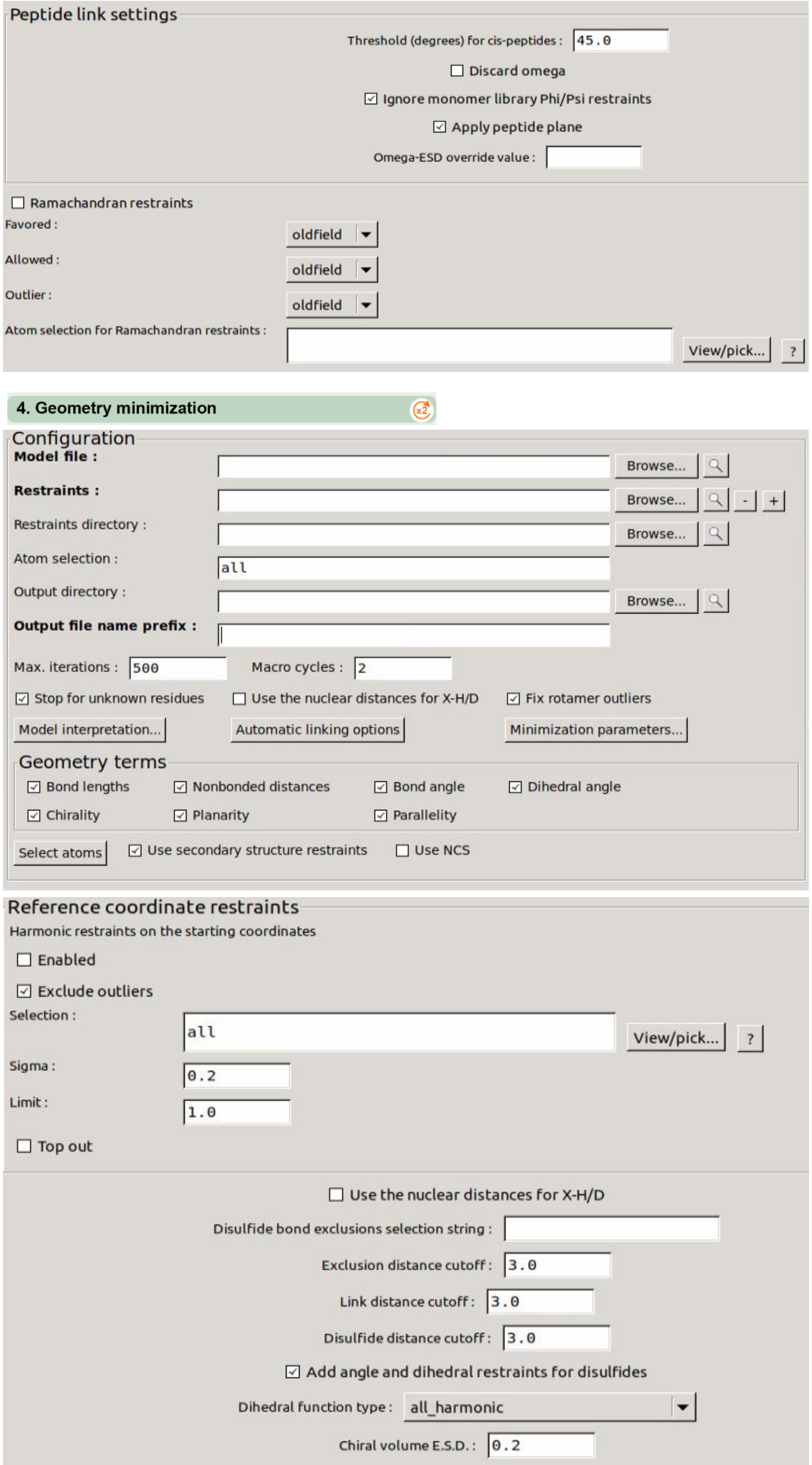

Peptide link settings
Threshold (degrees) for cis-peptides :
45.0
Discard omega
Ignore monomer library Phi/Psi restraints
Apply peptide plane
Omega-ESD override value :
Ramachandran restraints
Favored :
oldfield
Allowed :
oldfield
Outlier :
oldfield
Atom selection for Ramachandran restraints :
View/pick...
?
4. Geometry minimization
Configuration
Model file :
Browse...
Restraints :
Browse...
Restraints directory :
Browse...
Atom selection :
all
Output directory :
Browse...
Output file name prefix :
Max. iterations :
500
Macro cycles :
2
Stop for unknown residues
Use the nuclear distances for X-H/D
Fix rotamer outliers
Model interpretation...
Automatic linking options
Minimization parameters...
Geometry terms
Bond lengths
Nonbonded distances
Bond angle
Dihedral angle
Chirality
Planarity
Parallelity
Select atoms
Use secondary structure restraints
Use NCS
Reference coordinate restraints
Harmonic restraints on the starting coordinates
Enabled
Exclude outliers
Selection :
all
View/pick...
?
Sigma :
0.2
Limit :
1.0
Top out
Use the nuclear distances for X-H/D
Disulfide bond exclusions selection string :
Exclusion distance cutoff :
3.0
Link distance cutoff :
3.0
Disulfide distance cutoff :
3.0
Add angle and dihedral restraints for disulfides
Dihedral function type :
all_harmonic
Chiral volume E.S.D. :
0.2

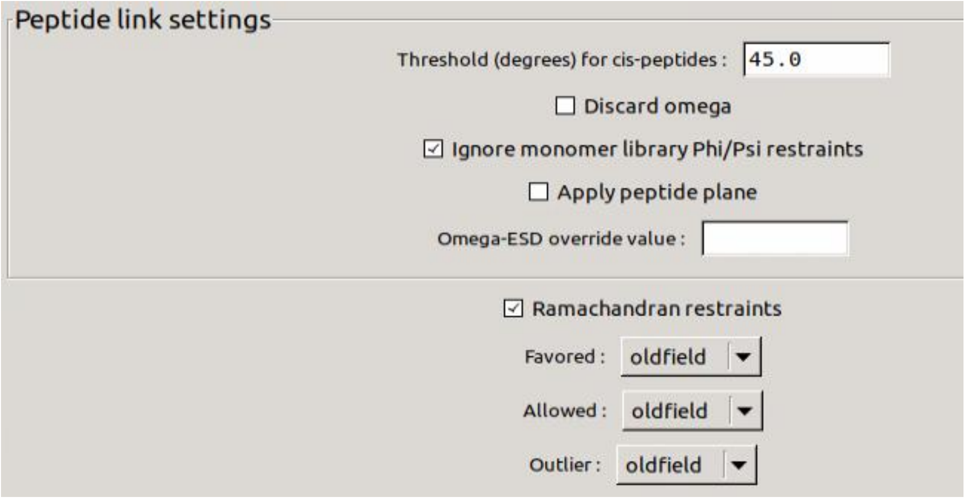
Peptide link settings
Threshold (degrees) for cis-peptides : 45.0
Discard omega
Ignore monomer library Phi/Psi restraints
Apply peptide plane
Omega-ESD override value :
Ramachandran restraints
Favored : oldfield
Allowed : oldfield
Outlier : oldfield

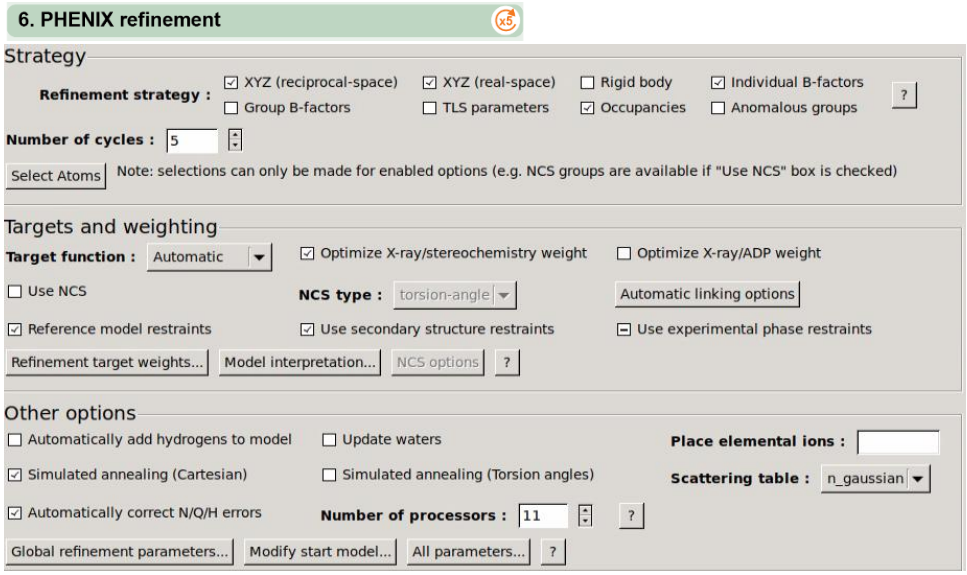
6. PHENIX refinement
Strategy
Refinement strategy :
XYZ (reciprocal-space)
XYZ (real-space)
Rigid body
Individual B-factors
Group B-factors
TLS parameters
Occupancies
Anomalous groups
?
Number of cycles : 5
Select Atoms
Note: selections can only be made for enabled options (e.g. NCS groups are available if "Use NCS" box is checked)
Targets and weighting
Target function : Automatic
Optimize X-ray/stereochemistry weight
Optimize X-ray/ADP weight
Use NCS
NCS type : torsion-angle
Automatic linking options
Reference model restraints
Use secondary structure restraints
Use experimental phase restraints
Refinement target weights...
Model interpretation...
NCS options
?
Other options
Automatically add hydrogens to model
Update waters
Place elemental ions :
Simulated annealing (Cartesian)
Simulated annealing (Torsion angles)
Scattering table : n_gaussian
Automatically correct N/Q/H errors
Number of processors : 11
?
Global refinement parameters...
Modify start model...
All parameters...
?

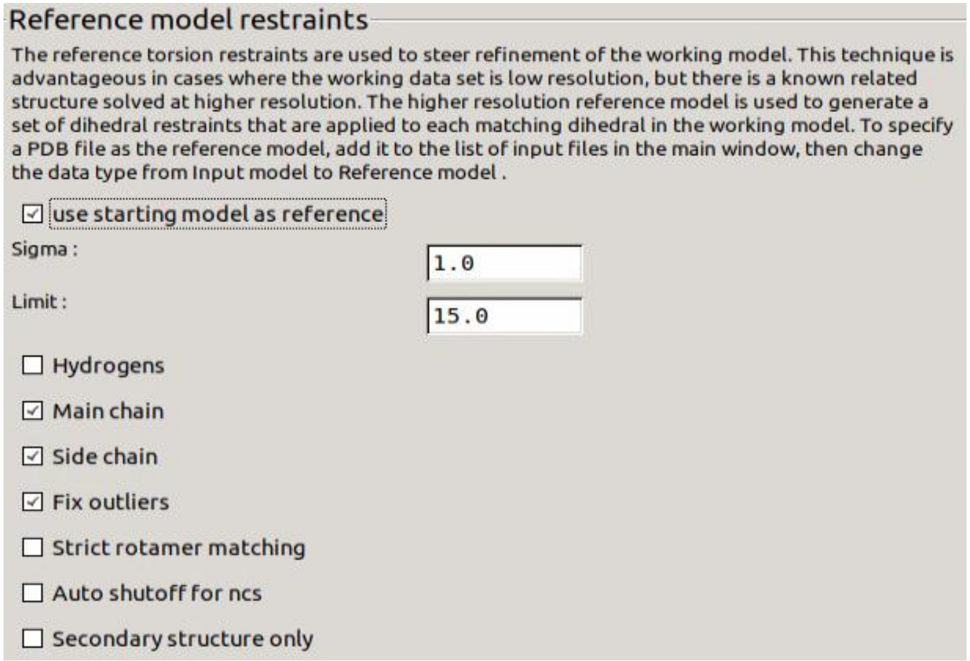
Reference model restraints
The reference torsion restraints are used to steer refinement of the working model. This technique is advantageous in cases where the working data set is low resolution, but there is a known related structure solved at higher resolution. The higher resolution reference model is used to generate a set of dihedral restraints that are applied to each matching dihedral in the working model. To specify a PDB file as the reference model, add it to the list of input files in the main window, then change the data type from Input model to Reference model .
use starting model as reference
Sigma : 1.0
Limit : 15.0
Hydrogens
Main chain
Side chain
Fix outliers
Strict rotamer matching
Auto shutoff for ncs
Secondary structure only

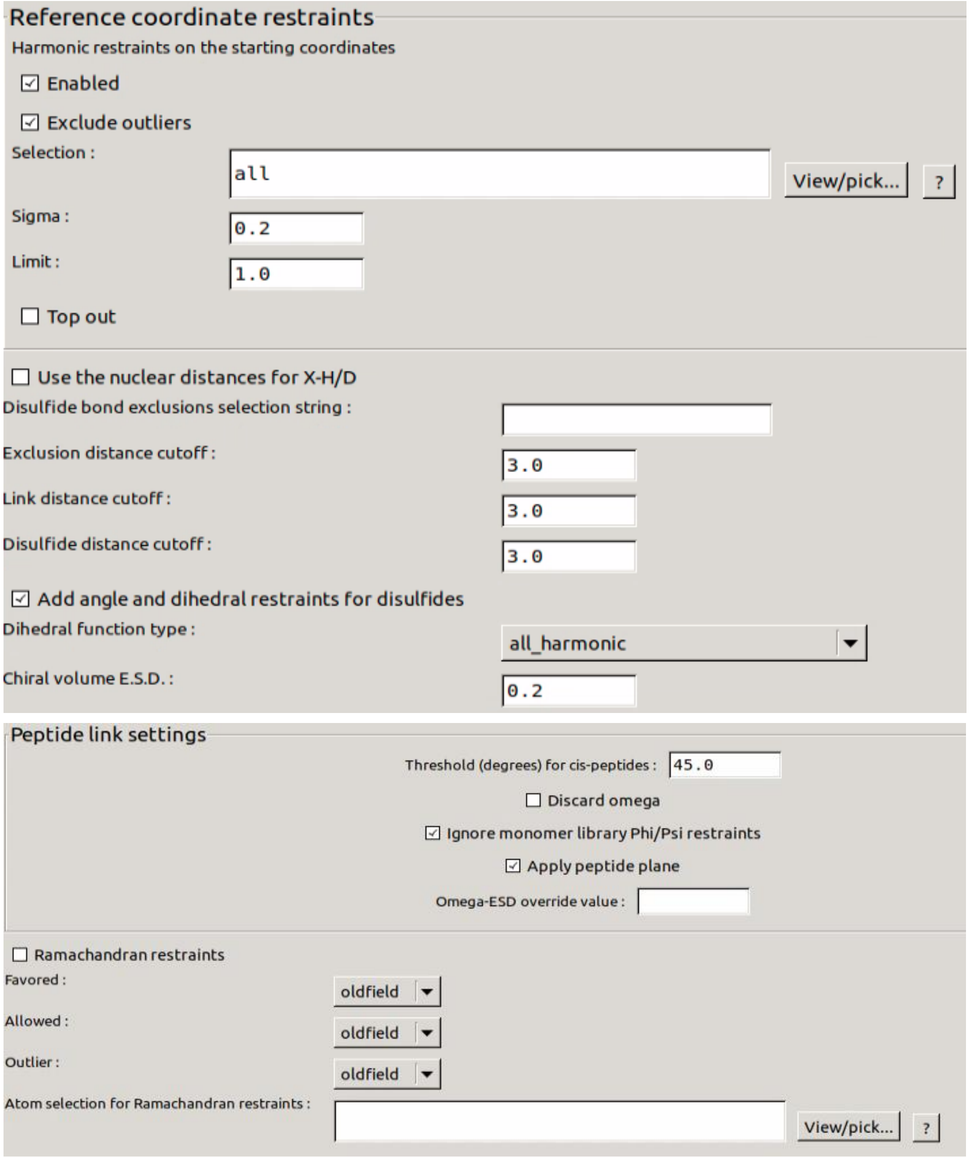
Reference coordinate restraints
Harmonic restraints on the starting coordinates
Enabled
Exclude outliers
Selection :
all
View/pick...
?
Sigma :
0.2
Limit :
1.0
Top out
Use the nuclear distances for X-H/D
Disulfide bond exclusions selection string :
Exclusion distance cutoff :
3.0
Link distance cutoff :
3.0
Disulfide distance cutoff :
3.0
Add angle and dihedral restraints for disulfides
Dihedral function type :
all_harmonic
Chiral volume E.S.D. :
0.2
Peptide link settings
Threshold (degrees) for cis-peptides :
45.0
Discard omega
Ignore monomer library Phi/Psi restraints
Apply peptide plane
Omega-ESD override value :
Ramachandran restraints
Favored :
oldfield
Allowed :
oldfield
Outlier :
oldfield
Atom selection for Ramachandran restraints :
View/pick...
?
